# To Break or to Brake Neuronal Network Accelerated by Ammonium Ions?

**DOI:** 10.1371/journal.pone.0134145

**Published:** 2015-07-28

**Authors:** Vladimir V. Dynnik, Alexey V. Kononov, Alexander I. Sergeev, Iliya Y. Teplov, Arina V. Tankanag, Valery P. Zinchenko

**Affiliations:** 1 Laboratory of intracellular signaling, Institute of Cell Biophysics, Russian Academy of Sciences, Pushchino, Russia; 2 Laboratory of bioenergetics, Institute of Theoretical and Experimental Biophysics, Russian Academy of Sciences, Pushchino, Russia; Mayo Clinic, UNITED STATES

## Abstract

**Purpose:**

The aim of present study was to investigate the effects of ammonium ions on in vitro neuronal network activity and to search alternative methods of acute ammonia neurotoxicity prevention.

**Methods:**

Rat hippocampal neuronal and astrocytes co-cultures in vitro, fluorescent microscopy and perforated patch clamp were used to monitor the changes in intracellular Ca^2+^- and membrane potential produced by ammonium ions and various modulators in the cells implicated in neural networks.

**Results:**

Low concentrations of NH_4_Cl (0.1–4 mM) produce short temporal effects on network activity. Application of 5–8 mM NH_4_Cl: invariably transforms diverse network firing regimen to identical burst patterns, characterized by substantial neuronal membrane depolarization at plateau phase of potential and high-amplitude Ca^2+^-oscillations; raises frequency and average for period of oscillations Ca^2+^-level in all cells implicated in network; results in the appearance of group of «run out» cells with high intracellular Ca^2+^ and steadily diminished amplitudes of oscillations; increases astrocyte Ca^2+^-signalling, characterized by the appearance of groups of cells with increased intracellular Ca^2+^-level and/or chaotic Ca^2+^-oscillations. Accelerated network activity may be suppressed by the blockade of NMDA or AMPA/kainate-receptors or by overactivation of AMPA/kainite-receptors. Ammonia still activate neuronal firing in the presence of GABA(A) receptors antagonist bicuculline, indicating that «disinhibition phenomenon» is not implicated in the mechanisms of networks acceleration. Network activity may also be slowed down by glycine, agonists of metabotropic inhibitory receptors, betaine, L-carnitine, L-arginine, etc.

**Conclusions:**

Obtained results demonstrate that ammonium ions accelerate neuronal networks firing, implicating ionotropic glutamate receptors, having preserved the activities of group of inhibitory ionotropic and metabotropic receptors. This may mean, that ammonia neurotoxicity might be prevented by the activation of various inhibitory receptors (i.e. by the reinforcement of negative feedback control), instead of application of various enzyme inhibitors and receptor antagonists (breaking of neural, metabolic and signaling systems).

## Introduction

It has long been known that the excess of ammonia (sum of NH_3_ and NH_4_^+^)can lead to lethargy, convulsions, ataxia and coma in patients with hepatic encephalopathy (HE) [1, 2]. On animal models of hyperammonemia, i. p. injections of lethal doses of ammonium acetate or NH_4_Cl may result in initial agitation and drowsiness, followed by clonic and tonic seizures, coma and animal death within 10–20 min [3–5]. The history of research in this field began in the 1890s from seminal works of Pavlov's group [6–8], who modified liver fistula developed in 1877 by Nikolai Eck [9] and described neurological alterations at portacaval shunting.

Since then a lot of attempts have been made to find effective methods of ammonia neurotoxicity prevention. Modern approaches in the treatment of patients with acute or chronic HE might be divided into two types of strategies, including: «ammonia lowering strategies» and «counteracting or neuroprotective strategies».

«Ammonia lowering strategies» are based on the suppression of ammonia production by gut flora (lactulose and antibiotics) and on the activation of ammonia consuming reactions (urea cycle and glutamine synthetase). These strategies represent core elements in the existing options of HE treatment during last fifty years [10–14]. Compositions of L-ornithine-L-aspartate, L-ornithine-α-ketoglutarate or L-ornithine-phenylacetate, etc. are often used to activate urea cycle and glutamine synthetase and to trap ammonia and glutamine [11–15].

«Counteracting or neuroprotective» strategies, which have been put forward over this period of time, were developed on cellular and animal models and have yet to be tested in clinical trials. Most of these strategies are based on the suppression of numerous cellular targets activated by ammonium ions, including glutamine synthetase, NADPH-oxidases or MAP kinases, nitric oxide synthases, numerous potassium channels and transporters, NMDA-receptor, etc. [16–32]. These «inhibitory» strategies are directed to prevent the consequences of ammonia neurotoxicity.

Astrocytes are considered as main targets and mediators of ammonia toxicity in the brain [29–35]. It is well known that at hyperammonemic conditions the suppression of glutamine synthesis in astrocyte may result in: partial animal survival [16, 17, 24], attenuation of increased extracellular potassium and amelioration of brain edema [24]. The inhibitors of lactate synthesis also may reduce edema and brain water content in bile duct ligated rats [23]. The blockade of NMDA receptors may also prevent or delay the death of animals, treated with lethal doses of ammonia [25, 26]. Systemic application of the inhibitors of NO synthesis display controversial results [27, 28].

No doubt that these «inhibitory» strategies, being aimed at the suppression of key elements of various signaling or metabolic systems, should have some restrictions in their application for ammonia neurotoxicity prevention. The applying of such inhibitors and antagonists in clinical practice apparently may be limited by their side effects.

Beside well defined alterations in metabolic and signaling systems of astrocytes [29–35], prominent changes in cooperation of different neurotransmitter systems are also observed under hyperammonemia. Excessive activation of NMDA-receptors [25, 26] and transformation of GABAergic tone [34, 35] in neural networks may also involved in the pathogenesis of HE at acute liver failure (ALF). Molecular mechanisms underlying these processes are poorly studied yet.

In the beginning of 70^th^ it was shown that, ammonia may block GABA and glycine mediated neuronal synaptic transmission (depression of postsynaptic inhibition), resulting in disinhibition and alteration of cortical functions [36, 37]. Long lasting application of ammonia slowly suppressed and then transformed hyperpolarizing GABA and glycine mediated action into depolarizing [38]. It was assumed, that observed «disinhibition phenomenon» (i.e. diminished GABAergic tone) may explain acute convulsant action of ammonia [39] and possible involvement of GABA(A) receptors blockade in myoclonus and convulsive seizures [40]. GABAergic neurosteroids (i.e. «endogenous benzodiazepines») are also considered as important factors, contributing to altered GABAergic tone at ALF and acute hyperammonemia. The rise in neurosteroids and GABAergic tone, according to some authors, may result in the development of brain edema and coma [34] and in the alterations of NMDA and GABA-dependent transmission [41, 42]. According to latest data, alteration of GABAergic transmission at hyperammonemic conditions may be realized on the basis of alternative mechanism. Ammonia is regarded as an agent, which may impair astrocyte potassium buffering, with following depolarizing action of GABA, neuronal network disinhibition and seizures [35].

The studies of ammonia toxicity on neural circuits are very limited and contradictory. It was shown only in recent time [43], that ammonia induces robust activation of neuronal network grown on microelectrode arrays, causing an increase of neuronal network activity and implicating NMDA receptors. Inhibitors of astrocyte glutamine synthesis or of NMDA receptors prevented network activation and protected these neuronal circuits against dysregulation produced by NH_4_Cl [43].

Our preliminary results, obtained on neuronal and astrocyte cocultures in vitro, show that ammonia invariably accelerates and transforms (unifies) neuronal network firing patterns implicating glutamate ionotropic receptors [44].

The main objectives of present work are to demonstrate that:

– some critical concentrations of ammonium ions may create bursting regimes combined with the acceleration of neuronal networks firing, implicating ionotropic glutamate receptors, but having preserved direct mode operation of GABA and glycine receptors and the activities of group of inhibitory metabotropic receptors;
– amplification of feedback control in the networks, based on reinforcement of functioning of various inhibitory receptors in the system, may result in suppression of networks hyperactivation, and might be used as the background in the search of new methods of pharmacological correction of hyperammonemic state.

## Materials and Methods

All animal studies were performed in accordance with the legal requirements and were approved by the Animal Ethic Committees of both institutes (Institute of Theoretical and Experimental Biophysics and Institute of Cell Biophysics, Russian Academy of Sciences).

### Cell culture preparation

Cell co-cultures of hippocampal neurons and astrocytes isolated from brain of newborn Sprague-Dawley rats (1–3 days old) were used in experiments in accordance with [45, 46]. All animal studies were approved by the Animal Ethic Committees of both institutes. The hippocampuses from neonatal Sprague-Dawley rats were dissociated with clippers and then incubated for 2 min in cold Hank’s Balanced salt solution (HBSS) containing Ca^2+^ and Mg^2+^. Supernatant was removed with pipette. 2 ml trypsin (0.1% in the Ca^2+^–Mg^2+^-free Hank’s solution) was added to pellet to cover whole tissue. The preparation was incubated in B27-supplemented neurobasal medium for 15 min at the 37°C with constant mixing on the thermo shaker at the 600 rpm. Then, trypsin was inactivated by equal volume of cold embryo serum, and preparation was centrifuged at 300×g for 5 min. To remove trypsin, cells were doubly centrifuged in the neurobasal medium. Then, cells were resuspended in this medium with addition of glutamine (0.5 mM), B-27 (2%) and gentamicin (20 g/ml). 200 μl suspension was put in glass ring with internal diameter of 6 mm standing on the round coverslip of 25 mm diameter (VWR International) covered by poly-l-lysine (one hippocampus to five glasses). Preparations were put in the CO_2_-incubator at the 37°C for 5h for cells attachment. After cells attachment, the cell culture glass rings were removed. Cultured medium (2/3 volume) was replaced every 3 days. The density of plated cells was 15000 cells/sq·cm. The neuronal cell cultures at the ages 5–7 or 12–22 days in vitro (DIV) were used in most experiments.

The changes in Ca^2+^-fluorescence signal in some way follow the voltage trajectory of each burst waveform of individual cell ([47–49; but see [50]). In this way arising network regimes could also be effectively monitored and characterized by registering Ca^2+^-signals of all cells implicated in the networks.

### Perforated whole-cell patch-clamp recording technique

Membrane currents from neurons loaded with Fura-2 am were recorded at 26°C with an Axopatch 200 B amplifier (Axon instruments) in the perforated patch-clamp configuration using pore-forming antibiotics nystatin. Nystatin stock (30 mM in Me2SO; Sigma) was prepared before each experiment, and used for up to 6 h after preparation. Data were digitized by a Low-noise Data Acquisition System (Axon DigiData 1440A digitizer) with pCLAMP 10 software from Axon Instruments (USA).

### Fluorescence measurements

To measure intercellular Ca^2+^, and pH_i_ we used the Carl Zeiss Cell Observer on the basis of inverted motorized microscope Axiovert 200M with high-speed monochrome CCD-camera AxioCam HSm and with high-speed light filters replacing system, Ludl MAC5000. For Fura-2 excitation and registration, we used filter set 21HE (Carl Zeiss, Germany) with excitation filters BP340/30 and BP387/15, beam splitter FT-409 and emission filter BP510/90, objective lens Plan-Neofluar 10×/0.3, excitation light source HBO 103 W/2. Calcium responses were recorded with double wavelength fluorescent probe Fura-2. Neurons were loaded with the probe dissolved in Hanks balanced salt solution (HBSS) composed of (mM): 156 NaCl, 3 KCl, 2 MgSO_4_, 1.25 KH_2_PO_4_, 2 CaCl_2_, 10 glucose and 10 HEPES, pH 7.4, at a final concentration of Fura-2 5 μM at 37°C for 40 min with subsequent 15 min washout. L-arginine (0.2 mM) was included in the medium. Changes in intracellular pHi were registered with dual-excitation ratiometric pHi dye indicator carboxy SNARF-1. Dye loading was performed according to the method described in [51]. Reagents applications were made in a continuous flow of HBSS solution by means of a special perfusion system that allows a quick replacement of the bathing solution. The experiments were performed by using 2–5 coverslips from 2–3 different cell cultures. N represents the number of cells implicated into network. n—number of the experiments. Examining 20 ms, 200 ms and 1 s frame rates, we selected 1 s sampling interval to filter out fast calcium fluctuations, register significant fluorescence changes and to avoid photobleaching effects. Calcium increment (VCi = ΔCa/min) was determined as area under curve above baseline (resting) calcium level (ΔCa), per unit of time. The changes in Ca^2+^_i_ are presented as the 340/380 ratio obtained from time-lapse images after background subtraction. All imaging experiments were performed at temperature 28–30°C. Excel, ImageJ and Origin 8 software were used for data analysis, graphs creation and statistic processing.

N—number of neurons implicate into network. N_1_ –number of astrocytes implicate into network. n = 3–5 (if not indicated in the legends)–the number of repeats of the experiment. Discrimination between neurons and astrocytes was performed on the basis of short-term (25 s) application of 35 mM KCl. Synchronously active fast-responsive to KCl cells were recognized as neurons. Most of astrocytes responded to KCl by low amplitude Ca^2+^ signals, with a few s delay. Part of astrocytes was not responsive to KCl. Results are expressed as means ± SD or SEM. Statistical analyses were performed using Student´s test for group comparison or one-way analysis of variance followed by Bonferoni multiple comparison test (ANOVA). The method of adaptive wavelet transform was applied for spectral data analysis. The complex-valued Morlet wavelet was also used as the analyzing function [52].

### Reagents

Amino-3-hydroxy-5-methyl-4-isoxazolepropionic acid (AMPA), N-methyl-D-aspartate (NMDA), Bicuculline methochloride, L-NAME hydrochloride, Glycine, Domoic acid, (R)-(+)-Methanandamide, L-arginine, Acetyl-L-carnitine chloride, UK 14304, Telenzepine dihydrochloride (Tocris Bioscience, UK); Hank’s Balanced salt solution (HBSS), neurobasal medium, B-27 Supplement, fetal bovine serum, fetal calf serum (Gibco); penicillin-streptomycin solution (Dalhimfarm, Russia); 0.1% poly-l-lysine; L-glutamine, L-glutamate, NBQX hydrate, (+)-MK-801 hydrogen maleate, NAAG, Betaine monohydrate, L-carnitine hydrochloride, P-F-HHSiD (p-Fluorohexahydro-sila-difenidol hydrochloride), Methoctramine hydrate, Nystatin (*Sigma*-*Aldrich*, USA); KCl (Chimmed, Russia); embryo calf serum (MP Biomedicals, USA); 4% gentamicin (Dalhimfarm, Russia); versene (Paneco, Russia); Fura-2 AM (Invitrogen, USA).

All animal studies were performed in accordance with the legal requirements and were approved by the Animal Ethic Committees of both institutes.

## Results

### 1. Transformation of Neuronal Network Firing Produced by NH_4_Cl

#### Ammonium chloride effects are concentration dependent

**Fig 1:**
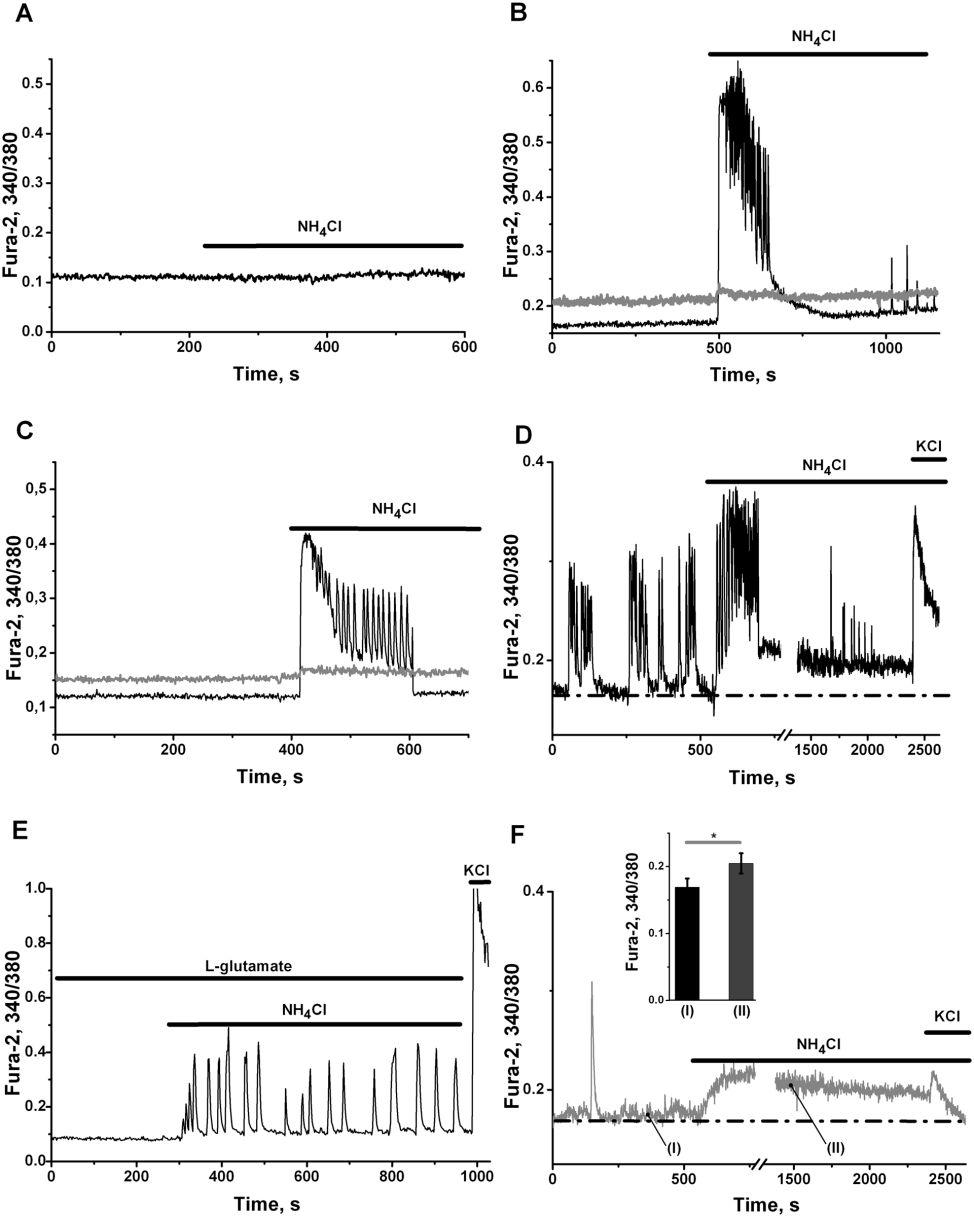
Ammonium chloride effects on neuronal network are concentration dependent. Here and later on calcium responses are measured by Fura-2 ratio. Records characterize individual neurons (black lines) and astrocytes (grey lines), i.e. representative cells of 90–95% cells implicated in the network. Thick horizontal black lines mark the periods of application of ammonium chloride (NH_4_Cl, 0.1–4 mM), L-glutamate (200 nM) and KCl (35 mM). **(A)** Application of 0.1 mM NH_4_Cl does not alter Ca^2+^ signals registered in representative cells: neurone (black line) and astrocyte (grey line). Neuronal culture 12 DIV. Number of monitored neurons in network N = 84 and number of astrocytes N_1_ = 47. Here is presented one of 12 experiments (n = 12). **(B)** Application of 1 mM NH_4_Cl induces one burst of high-amplitude Ca^2+^ oscillations in representative neuronal cell (black line) and does not alter significantly Ca^2+^ level in representative astrocyte (grey line). Neuronal culture 12 DIV. Number of neurons in network N = 96 and number of monitored astrocytes N_1_ = 52. Here is presented 1 of 3 experiments with evoked Ca^2+^ signal. Total number of experiments n = 20. **(C)** Application of 2 mM NH_4_Cl induces one burst of high-amplitude Ca^2+^ oscillations in representative neuronal cell (black line) wich is accompanied by some temporary elevation in astrocytic Ca^2+^-signal (grey line). Neuronal culture 15 DIV. Total number of neurons involved into network N = 102. Number of monitored astrocytes N_1_ = 43. Here is presented 1 of 2 experiments with evoked Ca^2+^-burst. Total number of experiments n = 10. **(D)** Application of 3 mM NH_4_Cl induces one burst of high-amplitude Ca^2+^ oscillations in representative neuronal cell (black line) with the rise in Ca^2+^ level in after burst period. Neuronal culture 14 DIV. Total number of neurons involved into network N = 91. Number of monitored astrocytes N_1_ = 47. Here is presented 1 of 3 experiments with evoked Ca^2+^-burst. Total number of experiments n = 12. **(E)** Induction of sustained Ca^2+^-oscillations in neuronal network by 4 mM NH_4_Cl. Record of representative neuron is presented. Neuronal culture 5 DIV. Total number of neurons in network N = 84. Here is presented 1 of 6 experiments with stable high-amplitude Ca^2+^-oscillations. Total number of experiments n = 10. 200 nM of L-glutamate was added before application of NH_4_Cl. **(F)** The record of Ca^2+^-signalling in astrocyte evoked by 3 mM NH_4_Cl. It corresponds to the experiment presented on Fig D. Application of 3 mM NH_4_Cl induces immediate rise of Ca^2+^ level to new steady state in representative astrocyte (grey line). Note the significant differences in the responses of neurons (Fig B) and astrocytes to depolarizing action of 35 mM KCl. Neuronal culture 14 DIV. Total number of neurons N = 91. Number of monitored astrocytes N_1_ = 47. Inserted black bars indicate the average amplitudes ± SD of intracellular Ca^2+^ level in 47 astrocytes recorded at time-points indicated. *P<0.05 is given for difference between both values.

In our experiments with dye loaded cells we usually can monitor network activity within 20–60 min. Application of 0.1–4 mM of NH_4_Cl (Fig 1) may produce different effects on network activity during this period of observation. Low concentrations of NH_4_Cl (0.1 mM) did not induce any remarkable changes in neuronal or astrocyte Ca^2+^-signaling (Fig 1A, grey and black lines correspondingly) in previously silent networks. After application of 1mM NH_4_Cl, some silent networks may generate one train (burst) of high-amplitude Ca^2+^-oscillations (Fig 1B, black line), without any changes in astrocyte Ca^2+^-signaling (Fig 1B, grey line). Such events we observed in 15% of cultures tested (3 of 20 cultures). Activation of neuronal networks by 2 mM NH_4_Cl also may evoke one burst Ca^2+^-oscillations (Fig 1C, black line), which may be accompanied by some temporal elevation in astrocyte Ca^2+^-signaling (Fig 1C, grey line). Higher concentrations of ammonium ions increase the probability of such transformation. In some spontaneously firing cultures (3 of 12 cultures) 3 mM NH_4_Cl may create one or several bursts of Ca^2+^-oscillations in neurons (Fig 1D) with visible sustained increase in intracellular Ca^2+^ in neurons (Fig 1D) and astrocytes (Fig 1F), which is preserved after cessation of Ca^2+^-oscillations.

This calcium rise over its initial resting level in representative astrocyte (Fig 1F) is in some correspondence with calcium increment observed in hippocampal astrocytes, after application of 1–5 mM NH_4_^+^/NH_3_ to hippocampal slices [33]. Nevertheless in our experiments registered changes in calcium concentrations in astrocytes are several times lower than corresponding values for neuronal cells during period of burst of Ca^2+^-oscillations (Fig 1D and 1F). Note also, that significant differences in the responses of neurons (Fig 1D) and astrocytes (Fig 1F) to depolarizing action of 35 mM KCl is observed in our cultures. Application of 4 mM NH_4_Cl may generate stable impulse-shaped Ca^2+^-oscillations in 60% (n = 10) of networks tested (Fig 1E).

**Fig 2:**
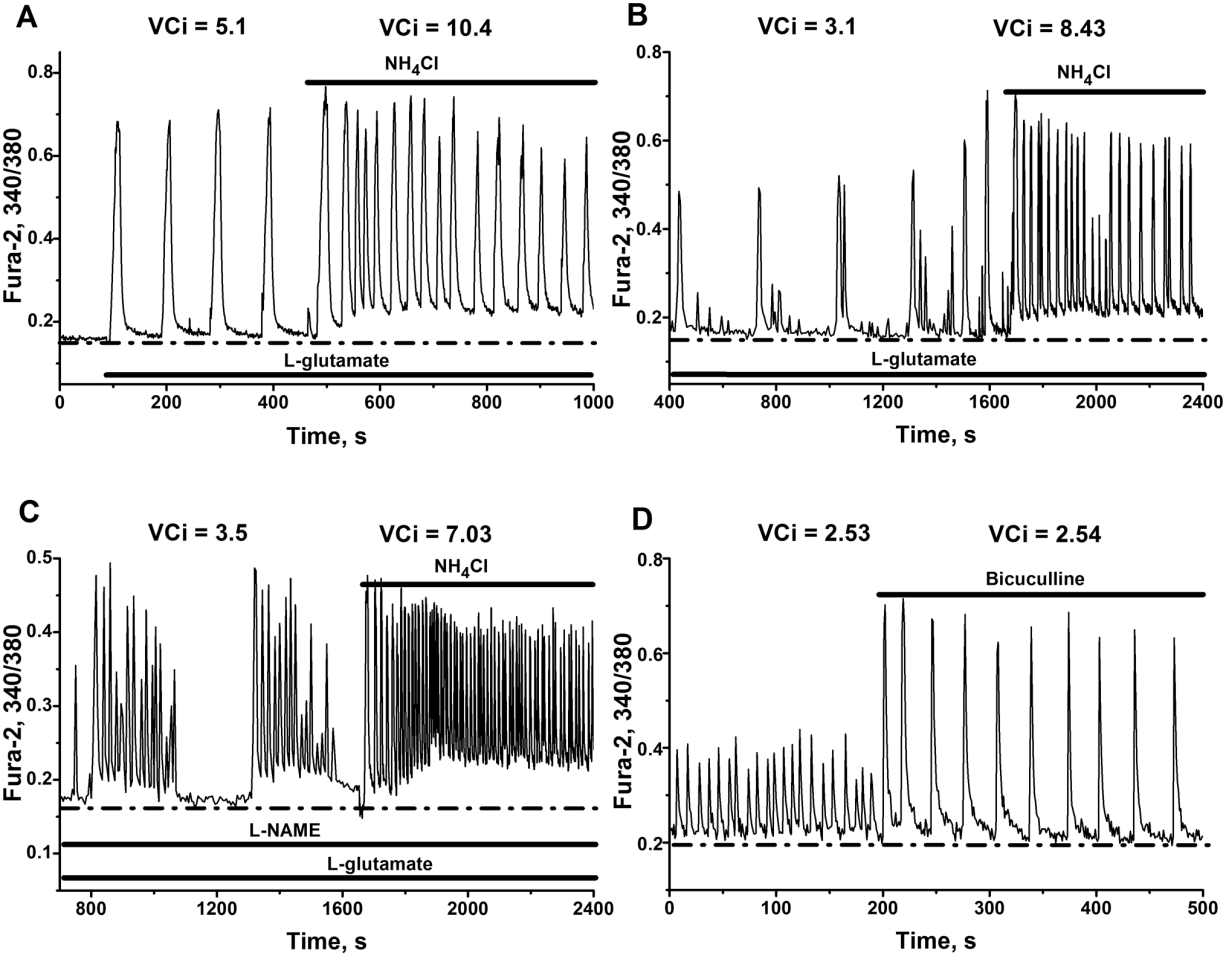
Transformation of simple and complex intracellular Ca^2+^-oscillations into high-amplitude impulse-shaped Ca^2+^-oscillations by NH_4_Cl or bicuculline. Neuronal cultures 12 DIV. Resting calcium level is outlined by dot-dashed lines. Calcium increment over resting level (VCi = ΔCa (a.u.)/min) is indicated on the Figures as VCi. All other abbreviations as on Fig 1. Total number of neuronal cells in networks are: N = 116, 132, 98, 110 for Fig A, B, C and D, correspondingly. **(A**–**C)** NH_4_Cl induces high-amplitude Ca^2+^-oscillations in representative cells. 200 nM of L-glutamate was added before application of NH_4_Cl. **(C)** The experiment was performed in the presence of 10 μM L-NAME and 200 nM of L-glutamate. **(D)** 10 μM of bicuculline evokes high-amplitude Ca^2+^-oscillations in spontaneously firing cell. Only parts of total records are presented on Fig B and C. Initial parts were omitted for simplicity.

**Fig 3:**
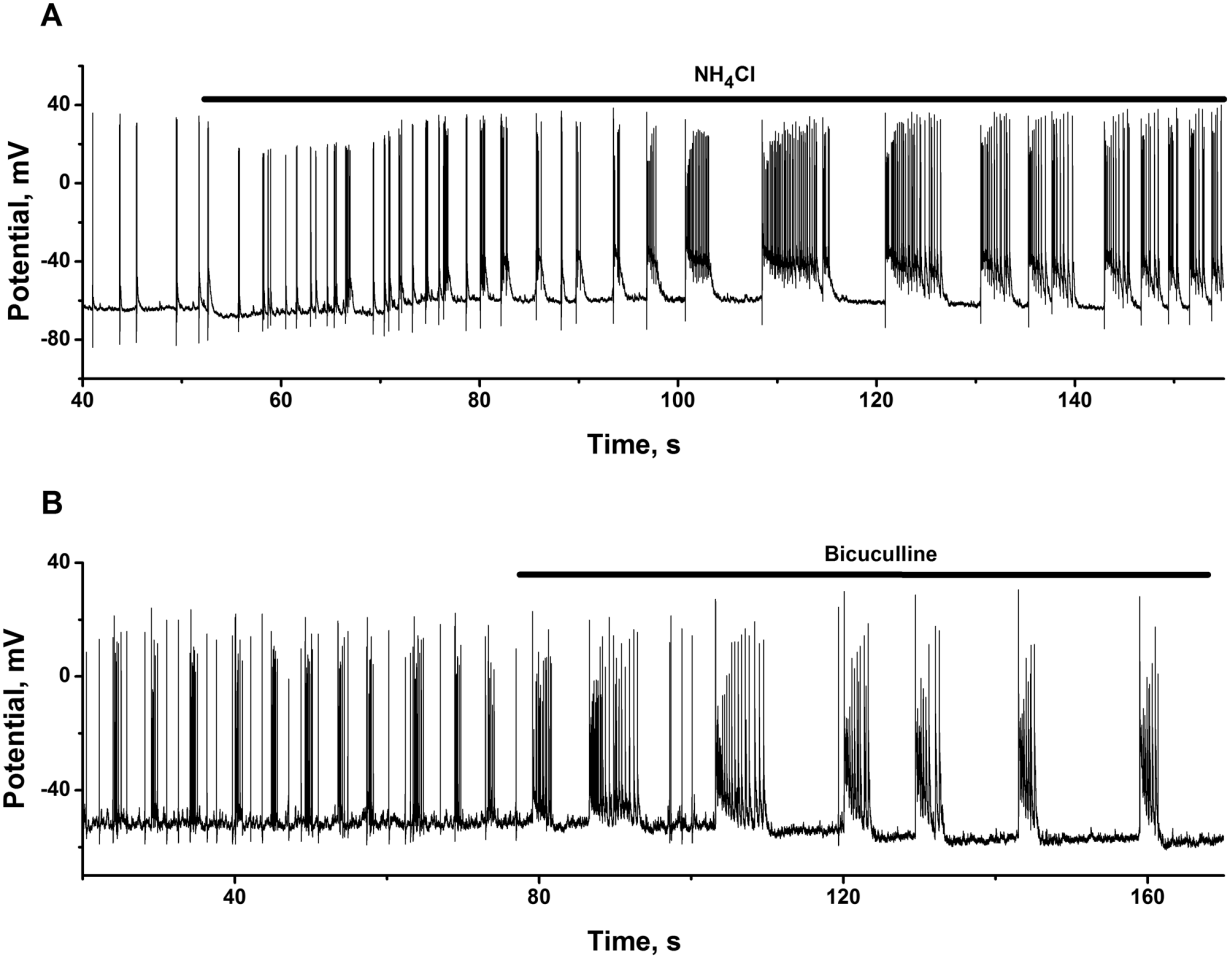
Typical discharge patterns of spontaneously firing cells and their transformation into strong burst firing produced by ammonium ions and bicuculline. Neuronal cultures 12 DIV. **(A)** NH_4_Cl (8 mM in the bath) causes the switching of tonic spiking to strong high-frequency burst firing, characterized by substantial depolarization of membrane potential at plateau phase of each burst. N = 97. n = 5. **(B)** Bicuculline (10 μM in the bath) transforms spontaneous bursting fluctuations of membrane potential into strong high-frequency burst firing. N = 89. n = 5. Here presented only parts of total records. Initial parts were omitted for simplicity.

NH_4_Cl, being applied at concentrations of 5–6 mM or higher, invariably accelerates Ca^2+^-oscillations in most of our neuronal networks. Nevertheless we selected for part of our experiments rather high concentration of NH_4_Cl equal to 8 mM (Figs 2A–2C and 3A), to have stable reproducible effects and to show that even at such conditions negative feedback control may still operate in the networks tested.

Our results about concentration dependent effects of ammonia are in some accordance with the results of experiments performed by Swartz and co authors on in vitro cortical neuronal networks grown on multielectrode arrays [43]. These studies have shown that application of 5 mM NH_4_Cl resulted in short-lived activation of network. Lower concentrations NH_4_Cl (0.5–2 mM) aroused delayed burst firing only after 24 hours of preincubation. Application of 10 mM NH_4_Cl in these experiments was characterized by stable immediate doubling of firing activity of network [43]. Apparently long-lasting preincubation is required for some kind of network sensitization to low doses of ammonium ions. We were interested in the study of acute toxic effects of ammonium ions.

#### NH_4_Cl unifies calcium oscillations and increases average calcium level. Comparison with bicuculline

Ammonia accelerates and unifies oscillatory regimes independently of initial state of network, (Fig 2A, 2B and 2C). These records are typical oscillatory regimes and characterize one representative cell of 93–96% of the cells implicated in the networks.

Neuronal networks may display 5 to 10 times changes in firing activity after application of of NH_4_Cl. The network presented on Fig 2A has initial period of Ca^2+^-oscillations equal to 100 s. After ammonia application this network switches into regime, which is characterized by Ca^2+^-oscillations with interspike intervals ranging from 3 to 7 s, soon after ammonia application, and to 10–12 s intervals after 30 min of recordings. Second and third examples describe the networks, which display complex spontaneous multimodal Ca^2+^-oscillations (Fig 2B and 2C). Ammonia transforms all these complex oscillatory regimes into impulse-shaped Ca^2+^-oscillations, with interspike intervals ranging from 2 to 6 s (Fig 2B) and from 10 to 15 s (Fig 2C) immediately and 10–15 minutes after ammonia addition, respectively.

Observed calcium increment over resting level per unit of time (VCi = ΔCa (a.u.)/min) may characterize cellular calcium homeostasis in such cells. Representative cells respond to ammonia by 2 to 3 fold rise in VCi (from: 5.1 to 10.4, 3.1 to 8.4 and 3.5 to 7.0), as it is indicated on Fig 2A, 2B and 2C.

Note, that in our experiments the transformation of network activity produced by GABA(A) receptors antagonist bicuculline is not characterized by the acceleration of Ca^2+^-oscillations and by rise in VCi (Fig 2D), as it produced by ammonia (Fig 2A–2C). Some authors suppose that diminished GABAergic tone or even depolarizing action of GABA [36–38] may explain acute convulsant action of ammonia [39, 40].

Observed difference in calcium handling between ammonia and bicuculline means that ammonia implicates different mechanism for activation of neural networks. Moreover drastic increase in neuronal calcium handling, which appeared at hyperammonemic conditions, represent substantial rise of calcium load in all cells implicated in network, in comparison with spontaneously firing cells or with cells affected by bicuculline.

#### Ammonia transforms discharge patterns of spontaneously firing cells into strong burst firing

Comparison with bicuculline. Fig 3 shows a whole cell current clamp recording of spontaneously firing neurons. These firing regimes may be classified into two distinct types:

- irregular sparse neuronal firing with single action potential (AP) or neuronal firing of a trains of AP without prominent membrane depolarization (Fig 3A; left side of record, before ammonia application);
- regular or irregular bursting of AP with visible (up to 3 mV) neuronal membrane depolarization (Fig 3B; left side of record, before bicuculline application).

Irrespective of initial dynamic state of the network, ammonia transforms silent or spontaneously active network into networks, generating: rhythmic bursting of AP, registered in selected cells after ammonia application (Fig 3A; right side of record), and fast synchronous impulse-shaped Ca^2+^-oscillations, observed in most cells in cultures studied (Fig 2A, 2B and 2C; right sides of records).

Fig 3A also shows, that the activation of spontaneously active networks by NH_4_Cl, leads to a slight depolarization of resting neuronal membrane potential (about 3–5 mV, dotted line), paired with the emergence of a regime, characterized by the generation of high-amplitude bursts, with 25–35 mV depolarization at plateau phase of potential.

It is well known, that burst-like firing regimes may be induced by various manipulations, including network «disinhibition», caused by the application of GABA(A) and/or glycine receptors antagonists [53, 54].

In our experiments ammonia strongly depolarize neuronal cells at plateau phase of potential (Fig 3A) in comparison with the action of bicuculline (Fig 3B). This drastic difference in the effects produced, again highlights the involvement of different mechanisms of networks activation by both agents.

It is obvious, that observed 25–35 mV increase in neuronal potential in the cells performing burst firing, determines raised calcium (and overall ionic) load in whole network at hyperammonemic conditions.

### 2. Characteristics of Network at Long Lasting Application of 5–6 mM NH_4_Cl

#### Appearance of «run out» cells

**Fig 4:**
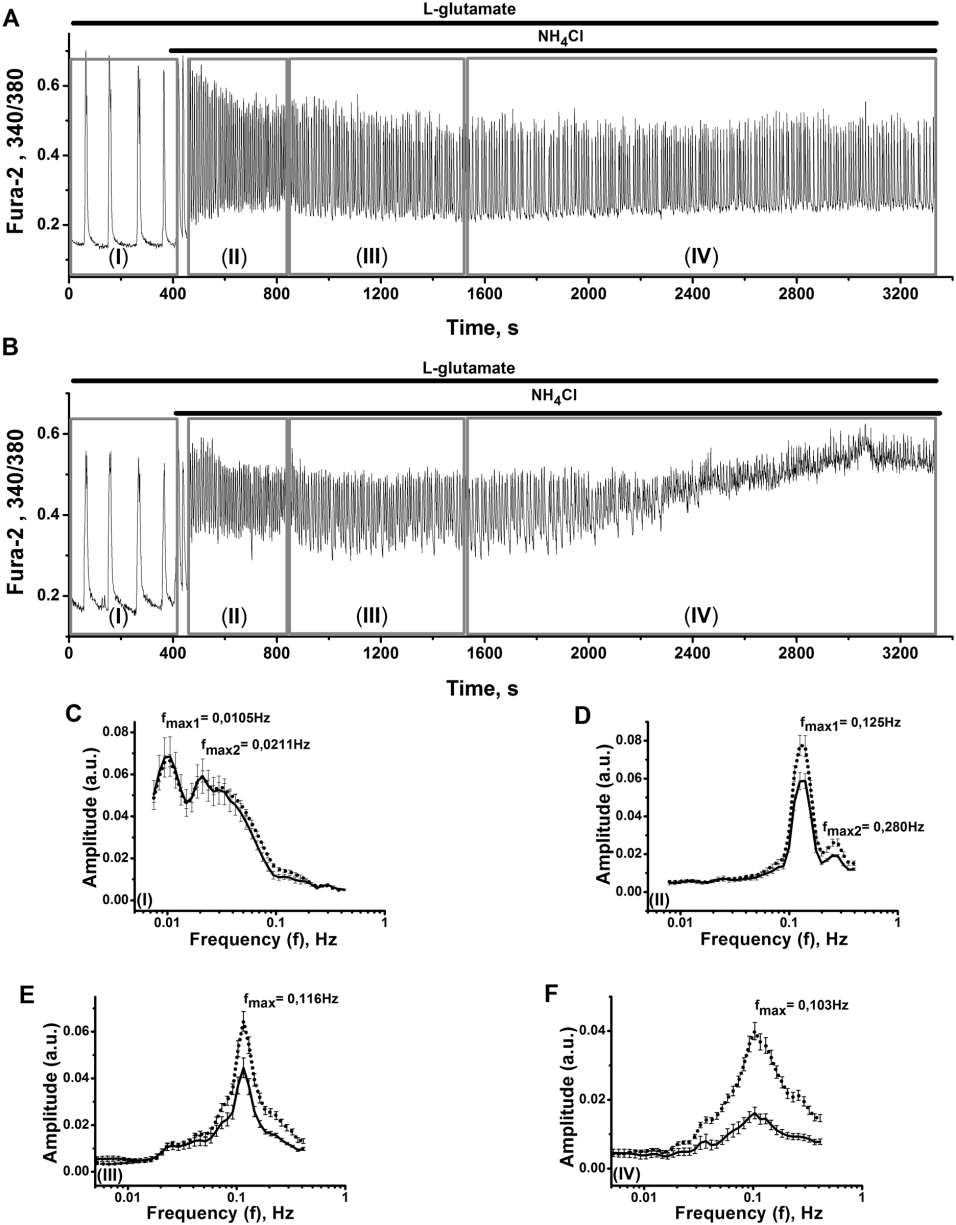
The records of Ca^2+^ oscillations (A, B) and Amplitude-frequency (A-f) spectral characteristics of Ca^2+^ oscillations (C–F) of two groups of neurons in network, observed after long lasting application of 6mM NH_4_Cl. Neuronal culture 14 DIV. Total number of cells involved into network is 123. 200 nM of L-glutamate was added before application of 6 mM NH_4_Cl. **(A)** The record describing typical response of one of 95% cells in the network (of representative cell). **(B)** The record characterizing one of 5% «run out» cells, which after time point 2000 seconds slowly moves to the state with enlarged Ca^2+^. **(C**–**F)** A-f spectra of two groups of cells (of typical and «run out» cells) are calculated for time intervals limited by boxes (I), (II), (III) and (IV) at Fig 4A and 4B. Dotted lines on A-f spectra correspond to 95% of cells with typical calcium response to NH_4_Cl (Fig 4A). Continuous lines correspond to 5% of «run out» cells (Fig 4B). SEM values are given for amplitudes deviations from average values and indicated by vertical bars. Maximal frequencies (f_max_) corresponding to maximal amplitudes are presented on the spectra.

Our networks display temporal changes in Ca^2+^-oscillations after application of NH_4_Cl, which are characterized by variations in periods and amplitudes of oscillations and by the appearance of several cells with steadily diminishing amplitudes of Ca^2+^-oscillations («run out» cells). First typical long-lasting firing regime in neuronal network activated by 6 mM NH_4_Cl is presented at Fig 4. Top record (Fig 4A) characterizes representative cell (one of 95% of 121 cells in the network). Lower record (Fig 4B) distinguishes one of 6 cells in the network («run out» cells), with different type of Ca^2+^-oscillations. In this kind of cells, average (for period of oscillations) Ca^2+^ level rises and the amplitude of oscillations steadily diminishes, starting from 2000 seconds of recording, in comparison with corresponding values of representative cell (Fig 4A).

#### Acceleration of oscillations and spectral characteristics of the system

Amplitude-frequency spectra (A-f spectra), calculated with the application of adaptive Wavelet transform method [52], for both populations of cells for four selected successive periods of recordings (Time boxes I–IV at Fig 4A and 4B) are presented at Fig 4C–4F. Both types of cells are characterized by similar forms of A-f spectra, independently of periods of their recordings. This may indicate that activities of «run out» cells are determined (controlled) by the dynamic state of network. Initial M-shaped frequency spectra (Fig 4C) characterizes bimodal (M-shaped) Ca^2+^-oscillations observed in all cells in the network (Fig 4A and 4B; Time box (I)). Here frequency f_max_ corresponds to maximum amplitude A_max_ at A-f spectra. Application of ammonium ions accelerates firing regime, rises f_max_ from about 0.01 Hz (Fig 4C; f_max_) to 0.13 Hz (Fig 4D; f_max_) soon after ammonia application. The frequency f_max_ diminishes to about 0.1 Hz after long lasting application of ammonium ions (Fig 4E and 4F; f_max_). Observed broadening of A-f spectra (Fig 4E and 4F) may indicate on diminished gain of negative feedback in the network [55]. At a moment we might only speculate that small population of «run out» cells, which may belong to one of subtypes of interneurons, lost its negative feedback control in network and this is reflected in broadening of A-f spectra.

#### Alterations in intracellular pH and temporal changes in Ca^2+^-oscillations

It is well known that besides its possible actions on groups of receptors and channels, ammonia may drastically alter intracellular pH in various types of cells. Perturbations in intracellular pH, produced by excess of ammonium ions in neuronal cells are well described [56] and characterized by fast initial pH overshoot, followed by slow accumulation of protons. Transition period may continue several minutes and results in intracellular pH fall on 0.2–0.3 units. This complex effect of ammonia may be confirmed by our experiments, which show that 6 mM of NH_4_Cl produce lowering of intracellular pH after initial pH overshoot. New steady state for protons in our network is established within 150–250 s (S1 Fig). It is known that ammonia evoked pH overshoot may result in intracellular Ca^2+^ spikes in astrocytes, accompanied by the release of glutamate [57]. Taking this into account, we might speculate that initial short-lived high-amplitude Ca^2+^-oscillations (Fig 4A and 4B; Initial part of Time box (II)) may be determined by combined action of released glutamate and of applied ammonia on NMDA-receptors of neuronal cells. Subsequent fall of intracellular pH might contribute in some way to declining of amplitudes and slowing of the frequency of Ca^2+^-oscillations (Time boxes II and III at Fig 4A and 4B). Appearance of «run out» cells (after 2000 s of recordings at Fig 4B) begins long after the transition of pH to new steady state level (after 300 s of recordings, S1 Fig). This means that elevated calcium level and low amplitudes of Ca^2+^-oscillations in «run out» cells may be determined by some other internal mechanisms of Ca^2+^-handling, not related directly to initial pH fall.

#### NH_4_Cl increases Ca^2+^-signalling in astrocytes implied in network

**Fig 5:**
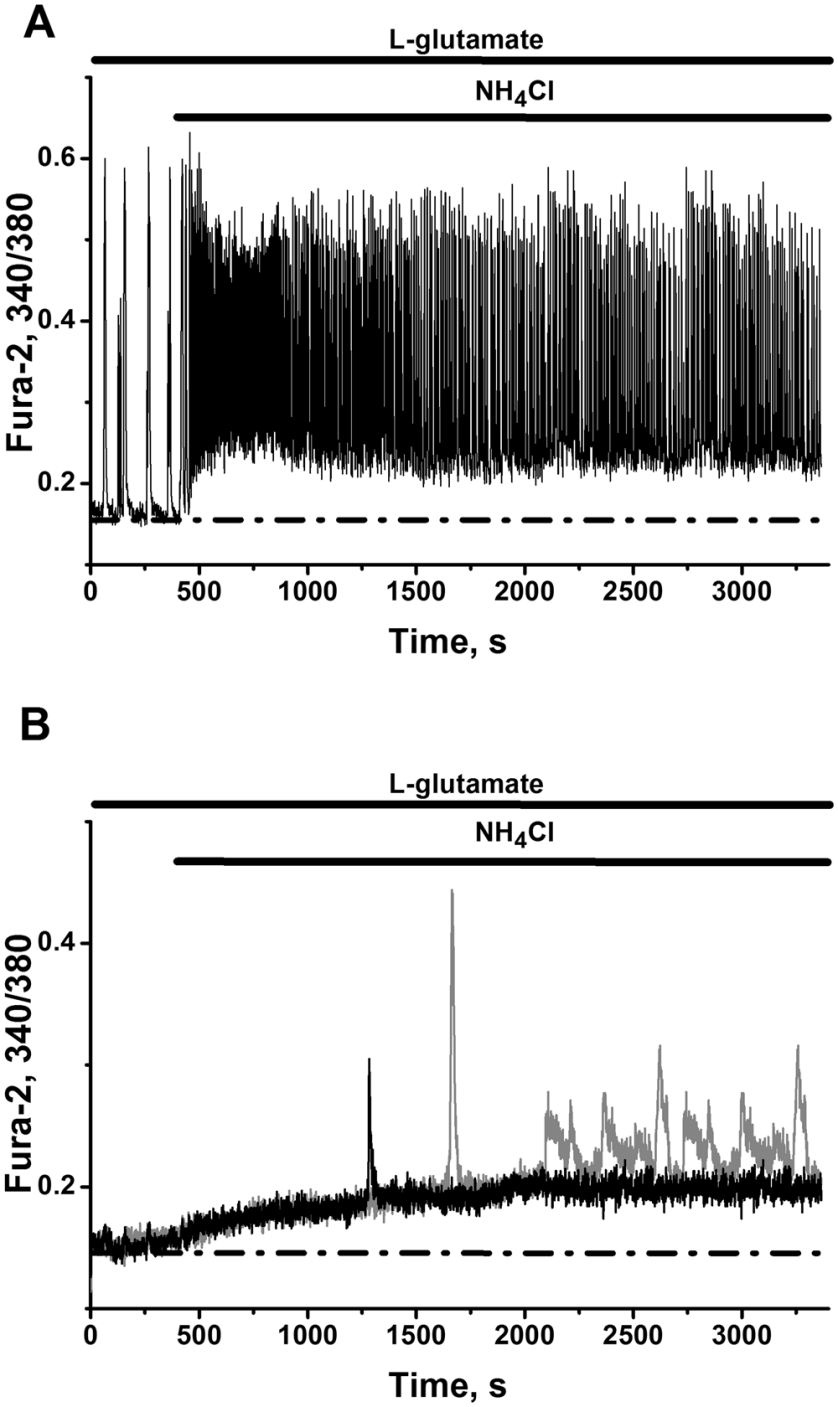
Comparative simultaneous recordings of Ca^2+^-oscillations in representative neuronal cell (A) and calcium signaling in two types of astrocytes (B) in network activated by 6 mM NH_4_Cl. Neuronal culture 14 DIV. Part of the experiment presented on Fig 4. All conditions as at Fig 4. **(A)** Recording of Ca^2+^ oscillations in representative neuronal cell (95% of cells) after application of NH_4_Cl. This is the repeat of the recording presented on Fig 4A. **(B)** Recordings of two types of responses of representative astrocytes are shown. NH_4_Cl slightly increases astrocyte Ca^2+^_i_ level with generation of solitary Ca^2+^ spikes in 44 of 67 cells (black line) and induce chaotic Ca^2+^ oscillations in 23 of 67 cells (gray line).

Beside acceleration of neuronal firing (Figs 2–4), rising of average for period of oscillations Ca^2+^-level in neurons (Fig 2) and the appearance of «run out» cells (Fig 4B), ammonia leads to appreciable increase of Ca^2+^ in one population of monitored astrocytes (66%) (Fig 5B, gray line) and in chaotic spiking phenomena in the rest of these cells (34%) (Fig 5B, black line). Taken together, these results and those presented earlier (Fig 1F), clearly show that sustained exposure to NH_4_Cl causes stable elevation and/or evoked chaotic oscillations in intracellular Ca^2+^ in astrocytes. However these changes of Ca^2+^ in astrocytes are substantially lower than average values of Ca^2+^ concentration observed in neuronal cells and there is no visible correlation between both processes.

**Fig 6:**
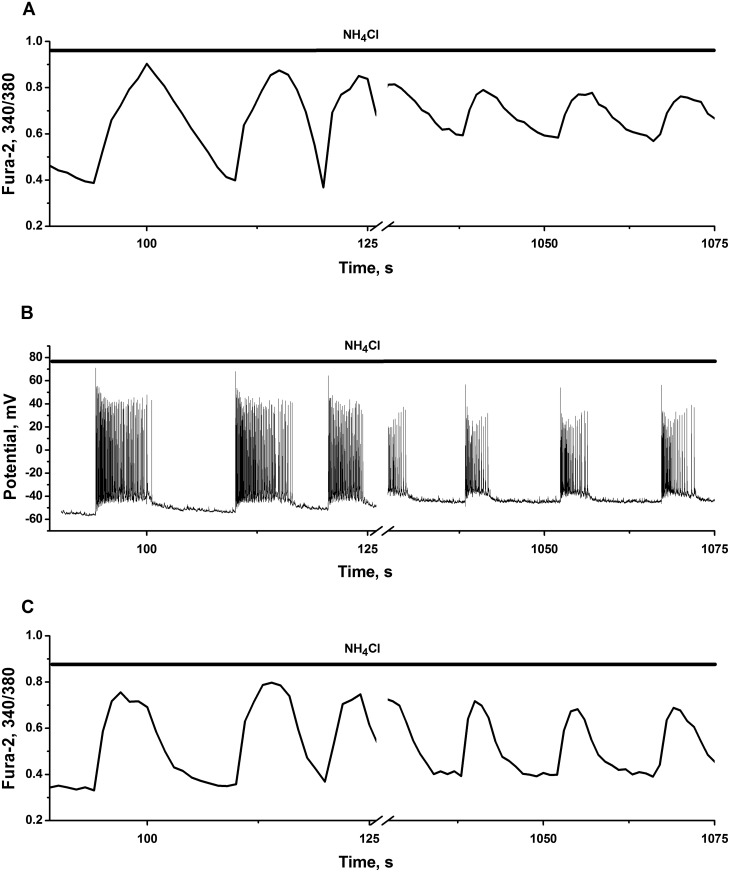
Simultaneous recordings of membrane potential and of Ca^2+^-oscillations in «run out» cell and representative cell in network activated by 5 mM NH_4_Cl. Neuronal culture 16 DIV. Total number of neuronal cells involved into network is 106. Gaps in the traces represent pauses in data recordings. Here are presented only parts of 3 records. Initial parts were omitted for simplicity. **(A)** Recording of Ca^2+^-oscillations in «run out» cell. **(B)** Recording of membrane potential in «run out» cell. **(C)** Recording of Ca^2+^-oscillations in representative cell (one of 95% cells monitored in network).

Fig 6 characterizes neuronal network activated by 5 mM NH_4_Cl and describes simultaneous recordings of Ca^2+^-oscillations (Fig 6A) and membrane potential (Fig 6B) in «run out» cell. For comparison the trajectory of Ca^2+^-oscillations of representative cell (96% of cells recorded in network) is shown at Fig 6C.

In the beginning of experiment, before ammonia application, short-term test of 35 mM KCl was applied to the network with the aim to discriminate the cells by the types of their responses. Some part of cells (10–12%) responded to KCl by fast calcium spikes (S2 Fig, trajectory 1). Representative cell demonstrated fast Ca^2+^ rise, slow Ca^2+^ decay till the end of KCl application and was characterized by fast Ca^2+^ fall after cessation of KCl application (S2 Fig, trajectory 2). Another representative cell, which is shown at Fig 4A, also demonstrated fast Ca^2+^ rise, kept some kind of calcium plato till the end of KCl application and also showed fast Ca^2+^ decay after finishing KCl application. This cell had Ca^2+^ trajectory, similar to trajectory 3, which is shown at S2 Fig.

On the contrary, «run out» cell responded to KCl application by slow Ca^2+^ rise. This process lasted till the end of KCl application and finished by slow Ca^2+^ decay after test cessation (S2 Fig, trajectory 4).

Comparing voltage and calcium trajectories we may observe some accordance between calcium responses of both types of cells to depolarizing KCl test and to AP bursts. In «run out» cell, time-period of Ca^2+^ rise is determined by the width of action potential burst and slow Ca^2+^ decay continues over all time of inter burst interval (Fig 6A and 6B). Representative cell (Fig 6C), demonstrates faster Ca^2+^ rise, keeps some kind of calcium plato till the end of potential burst and demonstrates fast decrease of intracellular Ca^2+^ within inter burst interval. Preliminary we might suppose that ammonia affects different populations of neurons implicating distinct mechanisms and separating most vulnerable groups of cells, like «run out» cells, that may belong to one the subtypes of interneurons.

### 3. Involvement of Ionotropic Receptors in the Control of Neuronal Network Accelerated by Ammonium Ions

It is widely recognized that ammonia toxicity is closely related to the activation of glutamate ionotropic NMDA-receptors (by yet unknown mechanism), due to fact that blockade of this receptor may delay or even prevent animal death at hyperammonemic conditions [25, 26]. Direct proof of such activation is still absent.

#### Synergy of NMDA and ammonia

**Fig 7:**
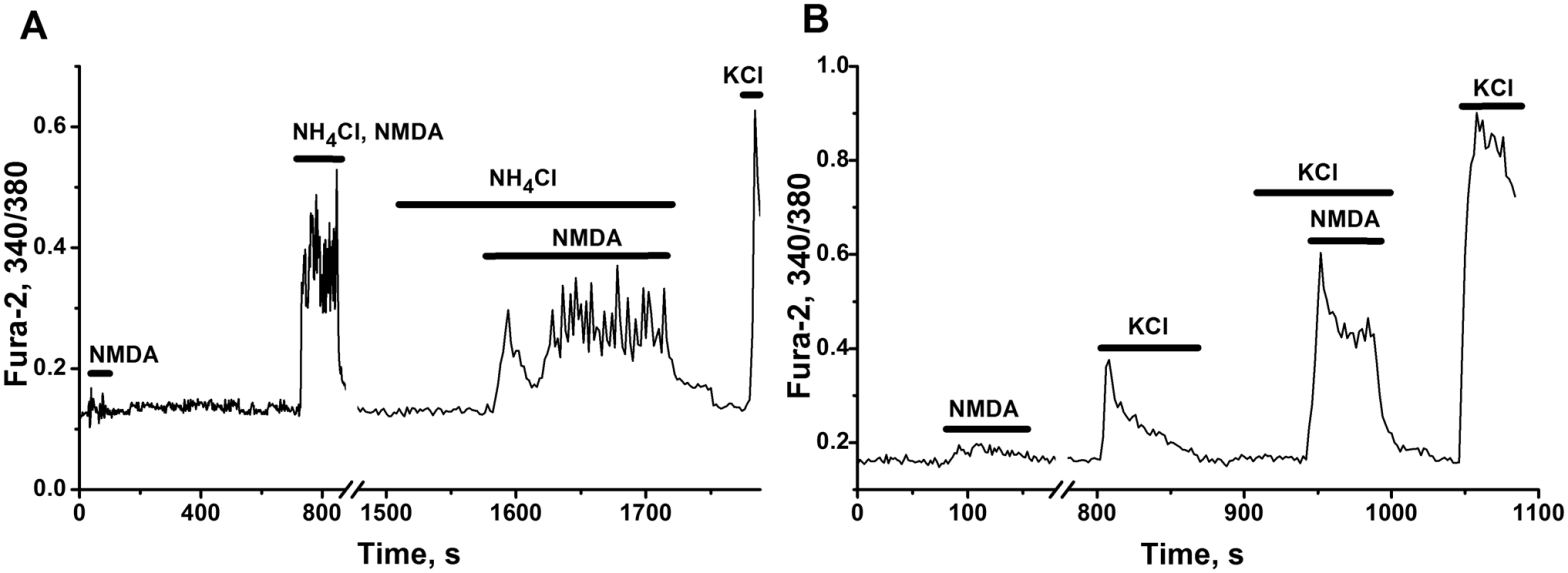
Synergistic action of ammonium ions and NMDA on changes in calcium concentrations in immature neuronal cells. Neuronal cultures 5 and 7 DIV. All other abbreviations and descriptions as on Fig 1. Gaps in the traces represent pauses in data recordings. **(A)** Combined synergistic action of NH_4_Cl (4 mM) and NMDA (10 μM) on cellular Ca^2+^ level, both of each separately cannot evoke Ca^2+^ signal and activate the cell. Culture 5 DIV. N = 66. **(B)** Neuronal Ca^2+^ responses to separate and combined action of NMDA (20 μM) and KCl (5 mM). Culture 7 DIV. N = 72.

Some authors defend the idea that network bursts may depend on recurrent excitation in the network [58]. In order to eliminate the influence of network activity on the effect of ammonia in individual neurons, we used also the culture of young cells (5–7 DIV), having no branched synaptic contacts (Fig 7). At 1 mM of Mg^2+^ in the incubation medium, low concentrations of NMDA or ammonium ions cannot activate silent neurons (Fig 7A). Combined application of 4 mM NH_4_Cl and 10 μM NMDA bring about to Ca^2+^-oscillations or bursting in the single cells (Fig 7A). These effects of mutual potentiation, demonstrate synergistic action of ammonium ion and NMDA on ionotropic NMDA receptors.

KCl (5 mM) depolarizes neuronal membrane to 10 mV and also potentiates the effect of NMDA (Fig 7B). This potentiating effect may involve another mechanism, based on removal of magnesium block of NMDA receptors.

#### Implication of NMDA and AMPA/kainate receptors in the activation of the neural network by ammonia

**Fig 8:**
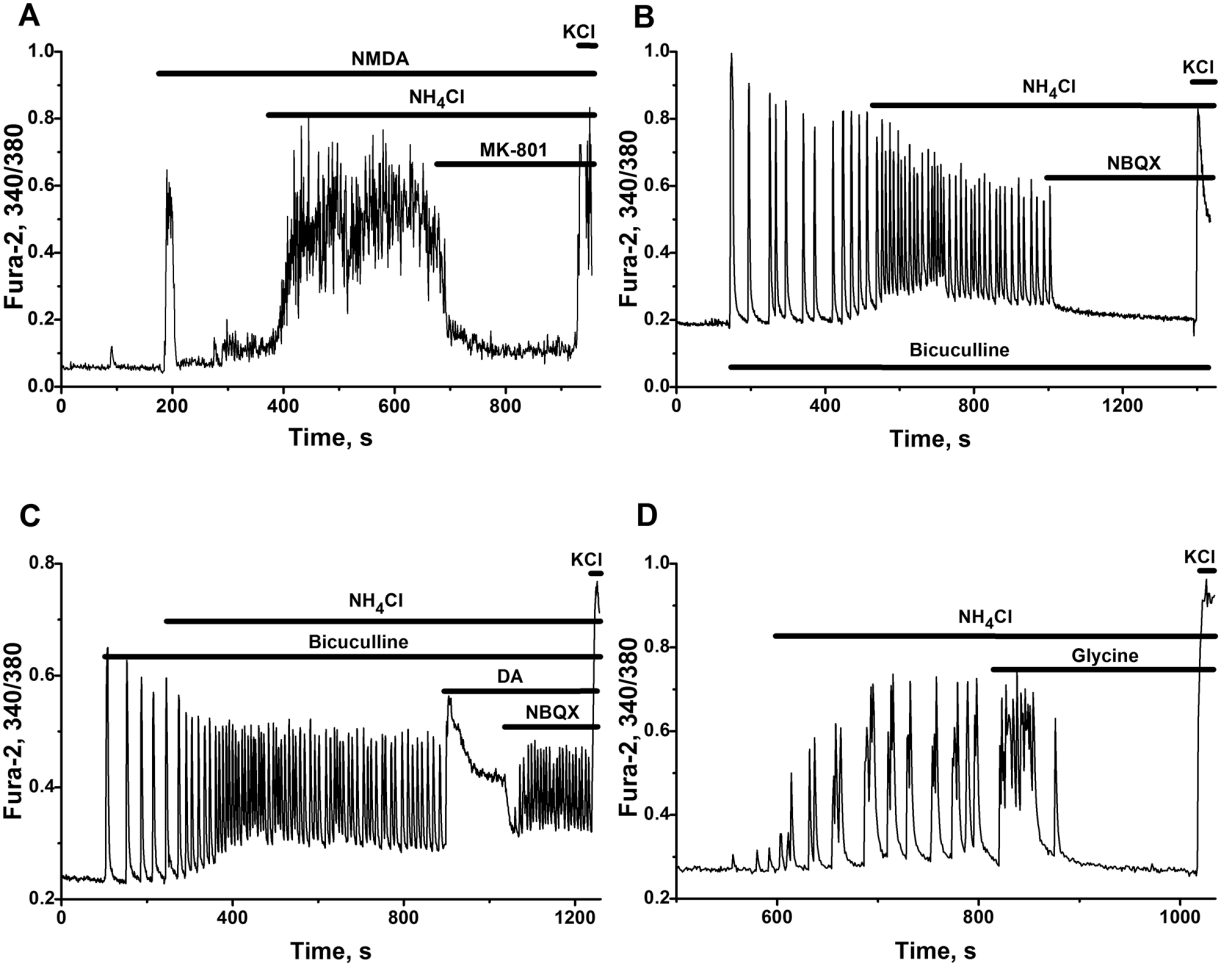
The involvement of ionotropic glutamate, GABA and glycine receptors in the activation of neural network by ammonium ions. Ca^2+^ responses of representative neurons (81% of cells on Fig A and 90–95% of cells on Fig B–D in networks monitored) were registered in cultures 12–16 DIV. All other abbreviations and descriptions as on Fig 1. **(A)** Suppressant effect of the antagonist of NMDA-receptors MK-801 (20 μM) on neuronal network accelerated by 8 mM NH_4_Cl in the presence of NMDA (10 μM). N = 97. **(B)** The antagonist of AMPA/Kainate receptors NBQX (20 μM) cancelled Ca^2+^ oscillations accelerated by 8 mM NH_4_Cl in the presence of GABA (A)-receptors antagonist bicuculline (20 μM). N = 124. **(C)** Excessive activation of AMPA/kainate receptors by domoic acid (DA, 200 nM) suppresses oscillatory regime in whole network disinhibited by bicuculline (20 μM) and further activated by NH_4_Cl (8 mM). Added NBQX (20 μM) renewed high-frequency oscillations, previously destroyed by DA. N = 132. **(D)** 100 μM glycine damped Ca^2+-^oscillations induced by 8 mM NH_4_Cl in previously silent network. N = 98.

The antagonists of NMDA and AMPA receptors 5S,10R)-(+)-5-Methyl-10,11-dihydro-5H-dibenzo[a,d]cyclohepten-5,10-imine hydrogen maleate (MK-801) (Fig 8A) and 1,2,3,4-Tetrahydro-6-nitro-2,3-dioxo-benzo[f]quinoxaline-7-sulfonamide disodium salt hydrate (NBQX) (Fig 8B) immediately suppressed neuronal network firing activated by ammonium ions. These results show that both types of glutamate receptors participate in the mechanisms of neuronal activation and bursting phenomena produced by NH_4_Cl.

Excessive activation of AMPA/kainate receptors by domoic acid (DA) suppresses oscillatory regimes in whole network (Fig 8C). This may indicate an important role of AMPA/kainate receptors in the modulation of the network activity accelerated by ammonia. The antagonist of AMPA/kainate receptors, NBQX, which acts competitively with respect to DA, diminished the gain produced by DA on AMPA/kainate receptors, restored the balance of positive and negative loops operating in the network and renewed high-frequency oscillations, previously destroyed by DA. This is reflected by reappearance of sustained Ca^2+^ oscillations in most cells in network (Fig 8C) and may confirm important role of AMPA/kainate receptors in the tuning of the network activity, accelerated by ammonium ions.

#### Operation of GABA(A)- and glycine receptors-dependent negative feedbacks in the presence of ammonium ions

Phenomena of fast acceleration of neuronal network firing produced by ammonia, is preserved in the presence of GABA(A) receptor antagonist bicuculline. Ammonia further boosts neuronal firing in the circuit disinhibited by bicuculline (Fig 8A). This clearly shows that possible «disinhibition phenomena» [36, 37], or expected «shunting» effect of chloride current [55, 58, 59], are not implicated in the observed immediate network overexcitation produced by ammonium ions.

The same conclusion is true for glycinergic negative feedback. Glycine—the principal transmitter released by glycinergic interneurons, may also participate, like GABA, in the control of neuronal firing [60]. It is known that long lasting application of ammonia slowly suppressed and then transformed hyperpolarizing GABA and glycine mediated action into depolarizing [38]. On the contrary, in our experiments, glycine, activating chloride influx, effectively slowed down network firing initially accelerated by ammonium ions (Fig 8D). According to these results we might suppose that both negative feedbacks may operate, at least on short time intervals, and are not modified substantially in the presence of ammonia excess.

As follows from Fig 8B and 8C, neuronal firing accelerated by ammonium ions, is still preserved in the network, in which GABA(A) receptors were switched off and AMPA/kainate receptors were partially suppressed. These results also may confirm the core role of NMDA receptors in the mechanisms implied in the activation of neural networks by ammonia. Thus, we can conclude that the activation of neural network by NH_4_Cl involves (as first step in complex process) the activation of NMDA and AMPA/kainate receptors and do not produce marked changes of neuronal resting membrane potential and modulation of GABA(A) and glycine receptors signaling.

### 4. Control of Neural Network Activity by Inhibitory Pre—and/or Postsynaptic Metabotropic Receptors is Preserved in the Presence of Ammonium Ions

It is well known that numerous pre and/or postsynaptic receptors are implicated in fine tuning and feedback and feed forward control of neural networks [61]. We have to expect that metabotropic: glutamate Type II (mGLURII), α2-adrenergic, m2,4-muscarinic, cannabinoid receptor type 1 (CB1) and D2,4-dopamine receptors, being involved in feedback control of specialized neural circuits in different brain areas, may also participate in the control of network activated by ammonium ions. Here we will focus only on some widespread negative feedbacks.

**Fig 9:**
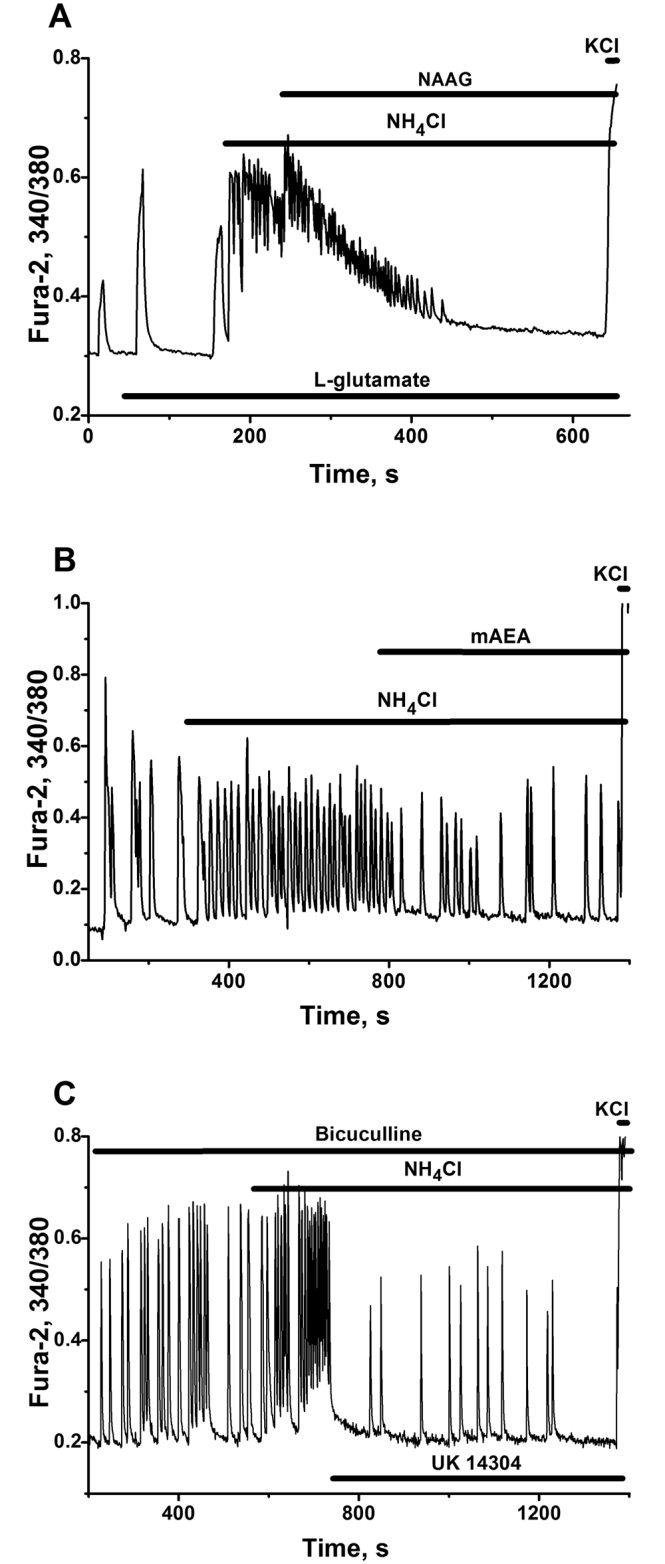
Effects of the agonists of inhibitory metabotropic receptors on the activation of neural networks by ammonium ions. Cultures 12–18 DIV. Responses of representative cells (90–95%) are presented. All other abbreviations and descriptions as on Fig 2. **(A)** Suppression of Ca^2+^-oscillations induced by 8mM NH_4_Cl after the application of 100 μM of metabotropic glutamate type II receptors agonist N-acetyl-aspartyl-glutamate (NAAG). 200 nM of L-glutamate was added before application of NH_4_Cl. N = 97. **(B, C)** Slowing down of Ca^2+^-oscillations in the network in the presence of 8mM NH_4_Cl by the agonists of α2-adrenoreceptors—UK14304 (100 nM, Fig B) and of cannabinoid CB1-receptors—mAEA (300 nM, Fig C). On Fig B the medium contained 10μM of bicuculline. N = 121 (for Fig B). N = 108 (for Fig C). Only part of total records presented on Fig C. Initial parts were omitted for simplicity.

In our experiments, the application of various agonists, implicating G_i_-protein coupled inhibitory pre and/or postsynaptic receptors, may markedly suppress the hyperexcitation of the neural networks, produced by NH_4_Cl (Fig 9), by damping the firing of most cells.

#### Functioning of mGLURII glutamate receptors

Activation of mGLURII glutamate receptors was shown to inhibit various neurotransmitters release in different brain areas [62, 63]. The agonists of these receptors have shown promise in the treatment of different brain disorders [64–66]. Natural neuropeptide neurotransmitter N-acetyl-aspartyl-glutamate (NAAG), activating mGluRII receptors, may be involved in negative feedback control of pyramidal cells ([67–70; but see [71]). As it shown on Fig 9A NAAG suppresses the hyperexcitation of neural network caused by ammonium ions. Inhibitory effect on neuronal firing is developed within several minutes. This result clearly indicates that mGLURII glutamate receptors participate in the control of neural networks even at excess of ammonium ions. This means that the agonists of mGLURII glutamate receptors potentially may be used in the treatment of acute episodes of HE.

#### Operation of CB1 cannabinoid receptors

It is known that cannabinoids, acting on presynaptic inhibitory receptors (CB1-type), may control excitatory glutamatergic [72] and inhibitory GABAergic synaptic transmission [73]. In our experiments cannabinoid (R)-(+)-Methanandamide (mAEA) counteract the effect of ammonium ions, slowing down the neuronal firing within several minutes (Fig 9B). We might speculate that due to combined action of cannabinoids on principal cells and interneurons, their inhibitory effect developed slowly and resulted in lowering of the frequency without damping the amplitude of calcium oscillations.

#### Implication of α2-adrenergic receptors in the control of networks operation

The modulation of the activities of pre/postsynaptic α2-adrenergic receptors may also be implicated in the control of neural networks [74, 75]. This kind of feedback control is widely used for the treatment of cognitive disorders [76, 77]. In our experiments suppression of network firing by the agonists of α2-adrenergic receptors 5-Bromo-N-(2-imidazolin-2-yl)-6-quinoxalinamine (UK 14304), is preserved in the presence of an excess of ammonia (Fig 9C).

All results presented above indicate that there is no marked influence of NH_4_^+^ on the activities of inhibitory metabotropic receptors in the networks affected by ammonium ions. This also means that pharmacological correction of hyperactivation of a neural network may be effectively managed using agonists of inhibitory pre/postsynaptic receptors of various types, including glycine and GABA receptors.

### 5. Protective Effects of L-Arginine and Methylamines

Among a large number of remedies, which have been tested in clinical practic and on animal models of hyperammonemia, L-arginine and the methylamines can be identified as the means combining ammonia lowering effects and neuroprotective activities. Below we demonstrate some impact of these substances on neural networks activated by ammonium ions.

#### Protective effect of L-arginine

**Fig 10:**
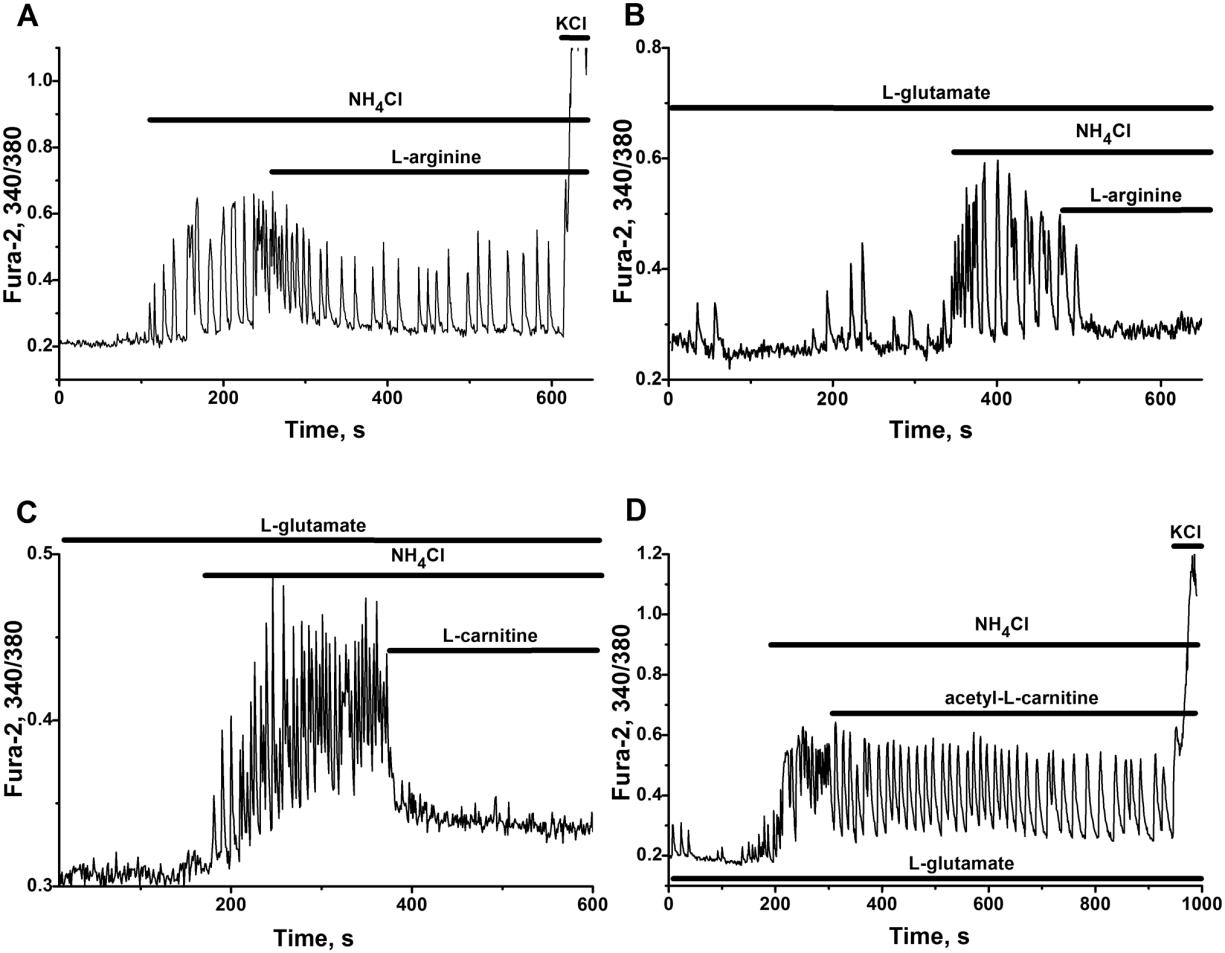
Suppressive and modulating effects of L-arginine, L-carnitine and acetyl-L-carnitine on neuronal networks activated by ammonium ions. Cultures 12–18 DIV. The records of representative cells (more than 90% of cells in culture) are presented. All other abbreviations and descriptions as on Fig 2. **(A, B)** Modulating (A) and suppressive (B) effects of L-arginine (10 mM). Typical responses of cells in few (Fig A; 20%) and in most (80%; Fig B) of cultures studied. n = 10. Culture 12 DIV. N = 89 (for Fig A). N = 106 (for Fig B). **(C, D)** Typical suppressant effect of L-carnitine (10 mM; Fig C) and modulatory effect of acetyl-L-carnitine (10 mM; Fig D) in most of cultures studied (75 and 80%, of cultures. n = 4 and n = 5 correspondingly). Culture 16 DIV. N = 111 (for Fig C). Culture 18 DIV. N = 126 (for Fig D). 200 nM of L-glutamate was added before application of 8 mM NH_4_Cl on Fig B, C, D.

L-arginine, being the substrate of NO-synthetases and intermediate of urea cycle in liver, also may work as weak α2-adrenoreceptors agonist in various types of cells [77, 78]. In neural network, activated by ammonium ions, the effect of L-arginine is more complex (Fig 10A and 10B), than immediate action of α2-adrenoreceptors agonist UK 14304 (Fig 9C). This effect of L-arginine seems to be dependent on combination of positive and negative feedbacks in corresponding network, which is determined by the expression of various proteins. High concentrations of L-arginine may produce slow decrease of frequency and amplitude of Ca^2+^-oscillations in 20% of cultures (Fig 10A) or an abrupt suppression of neuronal firing in 80% of cultures (Fig 10B).

#### Protective effects of methylamines

It has long been known, that some of methylamines: L-carnitine, choline, betaine, etc. may act as partial protectors of acute ammonia toxicity on animal models of hyperammonemia [5, 79, 80] and on cellular cultures *in vitro* [81–83].

**Fig 11:**
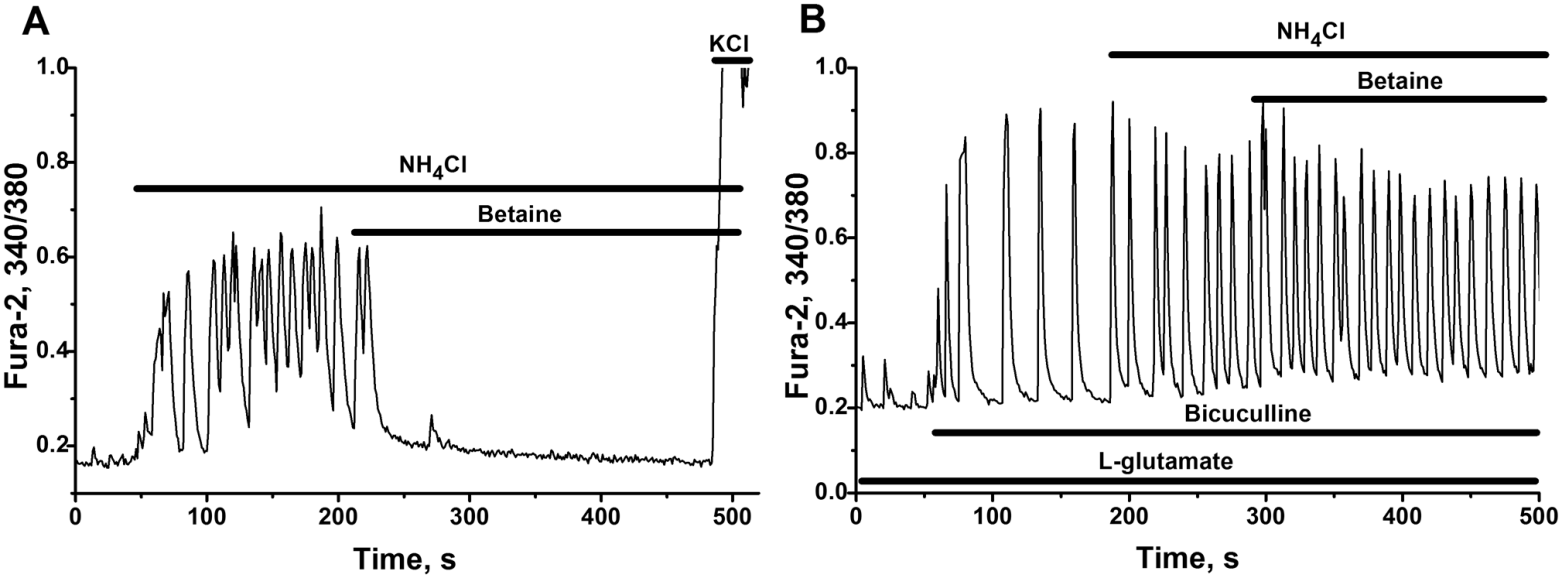
Inhibitory effect of betaine on the activation of networks by ammonium ions and its dependence on the operation of GABA(A)-receptors. Cultures 14 DIV. The records of representative cells. All other abbreviations and descriptions as on Fig 2. **(A)** Suppression of Ca^2+^-oscillations induced by 8mM NH_4_Cl after the application 10 mM of betaine. N = 136. **(B**) Disappearance of suppressive effect of betaine in the presence of GABA(A)-receptors antagonist bicuculline (10 μM). N = 124. 200 nM of L-glutamate was added before application of NH_4_Cl on Fig B.

Under our experimental conditions the application of acetyl-L-carnitine produced variable effects on networks, activated by NH_4_Cl. In most of studied cultures, acetyl-L-carnitine slowly diminished firing activity (Fig 10D). On the contrary, L-carnitine (Fig 10C) and betaine (Fig 11A) immediately and totally suppressed neuronal hyperactivation induced by ammonium ions. There is no additive effect of sequential applications of L-carnitine and betaine, or of acetyl-L-carnitine and betaine (S3 Fig). This may indicate some kind of similarity in the mechanisms of their actions.

It was supposed that all above mentioned methylamines have cholinergic muscarine-like action [81–83] and osmoprotective effect [61, 84, 85]. It was also shown that betaine promotes Ca^2+^-oscillations in adipocytes, acting via m3 muscarinic acetylcholine receptors [86]. Taking all this into account we might expect muscarine-like effect of some methylamines on neuronal circuits.

Observed suppressant effects of compounds tested, persist in the presence of the antagonists of m1, m2 or m3 muscarinic acetylcholine receptors (S4 Fig), though the forms of responses are modified and suppressant effects are developed with some delay. This clearly shows that the effects of methylamines on neural circuits may be associated not only with their possible action on muscarinic cholinergic receptors, but also with some other mechanisms.

#### GABA-like action of betaine

Fig 11A shows that betaine suppress NH_4_Cl-induced Ca^2+^ oscillations in neuronal network. The disappearance of betaine effect, observed in the presence of bicuculline (Fig 11B), means that the effect of betaine may be associated with the modulation of: the activity of GABA interneurons, or of GABA receptors, or of GABA content in medium. It is known that one of GABA carriers, operating in neurons and astrocytes (betaine-GABA transporter), is capable to transport betaine as well [87]. Taking this into account we can assume that betaine decrease GABA reuptake by astrocytes, which will cause the accumulation of GABA in extracellular environment and tonic activation of GABA receptors.

**Fig 12:**
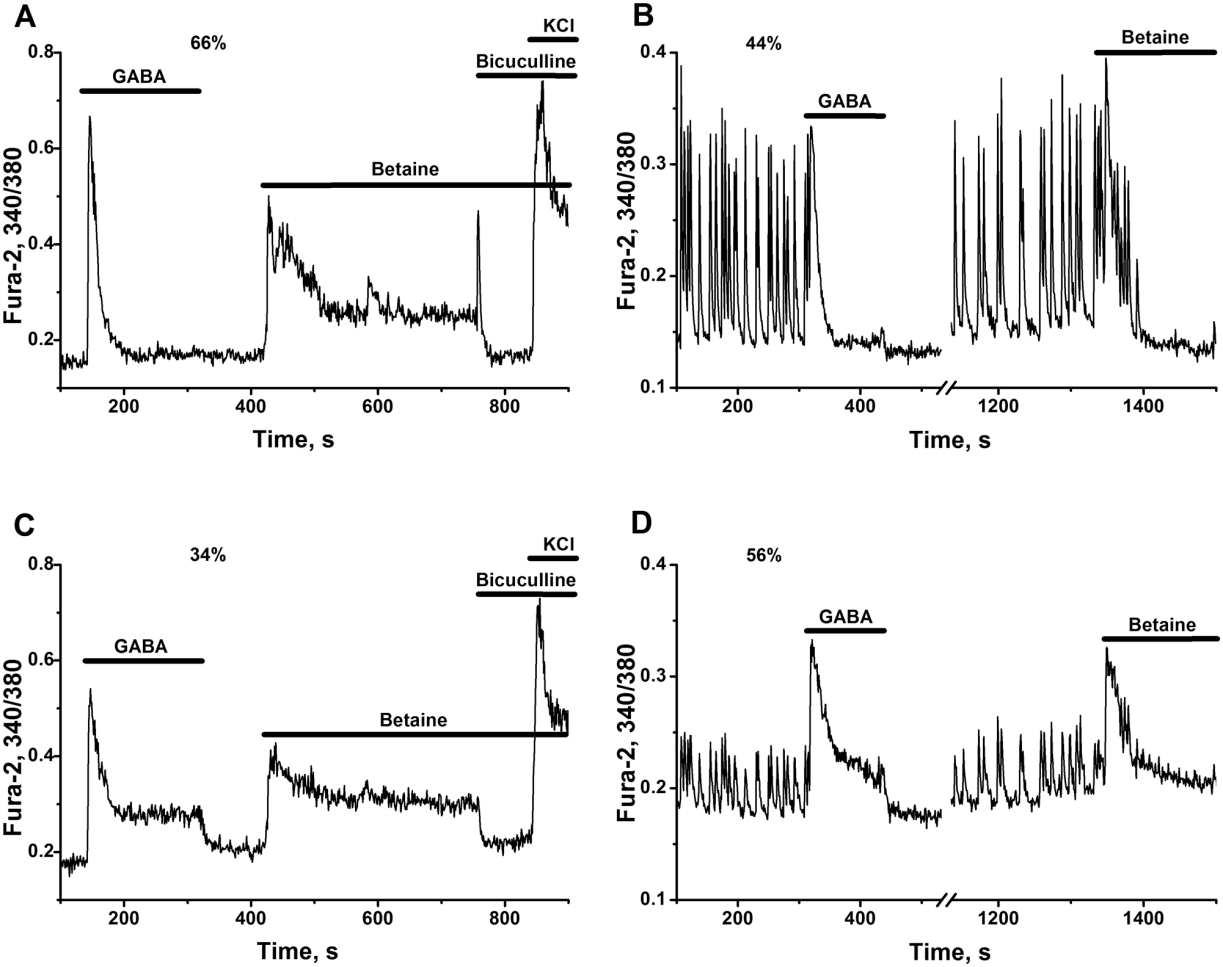
Similarity of the action of GABA and betaine on Ca^2+^-signaling in immature cells. Cultures 5, 7 DIV. The records of representative cells (to 60% of the cells on Fig A) and to 50% on Fig B). All other abbreviations and descriptions as on Fig 2. **(A, C)** Two types of Ca^2+^ responses to the application of GABA (5 μM) and one type of response to the action of betaine (10 mM) and suppression of betaine effect by bicuculline (2 μM) are observed in culture 5 DIV. N = 74. n = 3. **(B, D)** Inhibition of spontaneous Ca^2+^-oscillations in individual cells, observed in culture 7 DIV after addition of GABA and betaine. Two types of Ca^2+^ response to GABA and one type of response to betaine are observed in 20% of cultures in the cells with spontaneous activity. N = 36. n = 10. Here presented only parts of total records (Fig B, D). Initial parts were omitted for simplicity.

It is known that Cl^-^ concentration gradient is inverted in young neurons. Activation of GABA(A) receptors in this case causes neuronal depolarization and Ca^2+^ entry into cells. Using young neuronal cultures (5 DIV) with inverted operation of Cl^-^channels, we have shown that GABA and betaine induces an increase in Ca^2+^ in most of these neurons (Fig 12A and 12C). The responses to GABA allow to distinguish two types of cells which generate either Ca^2+^-spikes (Fig 12A) or the combination of Ca^2+^-spike and Ca^2+^-plateau (Fig 12C). The response to betaine looks like the second type of GABA response, in which Ca^2+^-spike and plateau phase are present (Fig 12A and 12C). Following application of bicuculline abolishes the effect of betaine.

In some neuronal cultures (7 DIV), in the cells with inverted operation of GABA(A)-dependent chloride channels, both compounds GABA and betaine effectively suppress spontaneous rhythmic activity (Fig 12B and 12D). This effect apparently may be related to known «shunting effect» of depolarizing chloride current [55, 58, 59] and inhibitory action of GABA and glycine on the neurons of newborn animals [88] or, on the contrary, to excessive amplification of positive feedbacks by depolarizing chloride currents. All these results once again support the hypothesis on GABA-like action of betaine.

Having all this as the background we might also suppose that observed suppressant effects of methylamines: L-carnitine, acetyl-L-carnitine, choline, betaine, etc. on neuronal networks hyperactivated by ammonia, may involve the modulation: of GABA concentration in the extracellular medium or/and of GABA receptors activity; muscarinic cholinergic receptors activities and/or some other mechanisms.

## Discussion

### Toxic level of ammonia and transformation of neuronal network activity

It is known that at acute episodes of HE in man registered level of ammonia in blood may rise up to 1.5 mM [89]. On animal models of hyperammonemia arterial and venous concentrations of NH_4_^+^ at its nadir may exceed 2–3 mM [4, 5]. At ALF brain ammonia concentration may be 3–6 times higher than in blood [16, 89] and in coma stages it may attain 5 mM [4, 5, 16, 89]. Such pathological concentrations are usually used in numerous acute experiments on isolated cells, slices or cell cultures.

Our neuronal networks (Fig 1A) do not respond immediately to low concentrations of NH_4_Cl (about 0.1 mM). Higher concentrations of NH_4_Cl (1–3 mM) may evoke temporal activation of networks, producing one or several bursts of Ca^2+^-oscillations (Fig 1B, 1C and 1D). It is known, that application of such concentrations of ammonia requires long time of expositions (up to 24 hours) to have reproducible stable effects on the activation of neuronal network in vitro [43].

Application of 5–6 mM NH_4_Cl immediately evokes neuronal potential burst firing and accelerates Ca^2+^-oscillations in all cells implicated in our networks (Figs 3A, 4 and 6). However, for part of experiments, we selected rather high concentration of NH_4_Cl equal to 8 mM, to have stable reproducible effects and to show, that negative feedback control in network is still preserved and operates at such conditions.

### Neuronal burst firing and high amplitude Ca^2+^-oscillations, calcium rise in neurons and astrocytes

Being activated by ammonia, neuronal circuits display stable burst-like firing regimes, characterized by 25–35 mV neuronal membrane depolarization at plateau phase of potential (Fig 3A), which defines high amplitude Ca^2+^-oscillations and substantial rise of average for period of oscillations Ca^2+^-level in all neurons implicated in network (Fig 2A, 2B and 2C). Significant increase in Ca^2+^-load is followed by the appearance of «run out» cells with steadily raised Ca^2+^-concentrations and diminished amplitude of oscillations (Fig 4B, time point after 2000 s). This may indicates that ammonia affects different populations of neurons, comprising distinct mechanisms and separating most vulnerable groups of cells, which may be responsible for further disturbances in network activities. Ammonia also increases astrocyte Ca^2+^-signalling, inducing elevation of intracellular Ca^2+^-level (Fig 1D), or sustained rise of Ca^2+^-level, combined with sporadic spiking or chaotic Ca^2+^-oscillations in various groups of cells (Figs 1D and 5B). These stages of astrocyte «syncytium» do not correlate directly with neuronal network activities. Both these processes are apparently interconnected, but direct links are not obvious yet, especially at acute action of ammonia on short time intervals. Such perturbations in intracellular Ca^2+^-homeostasis may be responsible for chain of events determining the progress of ammonia induced neurotoxicity, including overactivation of various Ca^2+^-dependent processes in both types of cells [22, 30, 31], accumulation of ROS, NO and dysregulation of NO-cGMP signaling pathways [25, 26], alterations in glutamatergic and GABAergic neurotransmission and cellular death [90].

### Implication of ionotropic glutamate receptors in ammonia induced neurotoxicity

Excessive NMDA receptors activation by ammonium ions is considered as a main contributor, leading to initial Ca^2+^ accumulation in neural cells [22, 25]. Blockade of NMDA receptors delays animals death at hyperammonemic conditions [22, 25, 26], what supports this notion.

In our neural networks blockade of NMDA receptors suppresses neuronal firing accelerated by ammonium ions (Fig 8A), what also corresponds to known results. Similar inhibition of network firing may also be produced by the blockade (Fig 8B) or, vice versa, by over activation of AMPA/kainite receptors (Fig 8C). Apparently this over activation AMPA/kainite receptors depolarizes most of the cells implicated in the network and switches the system into the state with high intracellular calcium. Taking all this into account we might suppose that AMPA/kainate receptors play important role in the tuning of the networks, accelerated by ammonium ions. Apparently some other mechanisms determining neuronal membrane depolarization at plateau phase of potential may also underlie corresponding over activation of neuronal NMDA receptors.

### Potential mechanisms of bursting

Burst-like firing regimes in the neurons of various types may be observed after substantial membrane depolarization produced by depolarizing currents; after the application of NMDA (AMPA) receptors agonists and of some potassium or of nonselective cation currents antagonists [91–96], or at activity dependent depolarizing action of inhibitory transmitter GABA [59]. Ammonium ions and trimethylamine may cause burst firing by inhibiting the medium and slow afterhyperpolarizations [97]. Burst-like regimes may also be induced by «disinhibition», caused by the application of GABA(A) and/or glycine receptors antagonists [53, 54]. However, in comparison with the effect of ammonia, «disinhibition» produced by bicuculline is not characterized: by substantial neuronal membrane depolarization at plato phase of potential (Fig 3B); by acceleration of Ca^2+^-oscillations and by rise in VCi (Fig 2D). Ammonia apparently implicates drastically different mechanism. Further studies, combined with mathematical modeling, seems to be required to determine the group of channels and transporters, which may be affected by ammonium ions and underlie burst firing under hyperammonemic conditions. At a moment we may only speculate that neural networks are robust with respect to bursting phenomenon. Several different configurations of current and receptor activities might be responsible for appearance of such regimes.

### Disinhibition phenomenon» and networks acceleration

Well-known suppression of GABA and glycine mediated responses by ammonia (i. e. «disinhibition phenomenon» [34, 37–40]) was used to explain acute convulsant action of ammonia [40] and the involvement of GABA(A) receptors blockade in myoclonus and convulsive seizures [34]. It was also shown that «shunting» inhibitory effect [58, 59] of depolarizing GABA-dependent chloride current may result in acceleration and stabilization of network firing [55]. Regarding possible «shunting» effect of chloride current, we have to assume that ammonium ions must set resting GABA chloride current potential within narrow limits of values to realize bidirectional «shunting» effect of depolarizing chloride current [55].

It is widely recognized that astrocytes play key role in the progress of HE, being considered as main targets and mediators of ammonia toxicity in the brain [29–35]. Beside «metabolic» toxic effects, resulting in deregulation of various metabolic and signaling systems, alterations in intracellular ion homeostasis in astrocytes is considered to be the second (if not first) hit in the development of acute HE. According to latest data astrocytes may also contribute to alterations of ionic homeostasis and «disinhibition phenomenon». As is known, ammonia may trigger elevation of sodium [98] and calcium [31, 33, 99] in astrocytes. Accumulation of ammonia in astrocytes may also impair astrocyte potassium buffering, with resulting accumulation of extracellular potassium, neuronal network disinhibition and the appearance of generalized clonic and tonic seizures [35].

Our in vitro neural networks, accelerated by ammonium ions, do not display «disinhibition phenomenon». Ammonia further boosts neuronal firing in the circuits disinhibited by bicuculline (Fig 8A). GABA, glycine and betaine effectively suppress the firing of networks accelerated by ammonium ions (Figs 8B and 11A). This may denote that: «disinhibition phenomenon» [36–40] is not realized in the network studied and do not contribute to the networks acceleration; direct or indirect inhibitory (transforming) effect of ammonium ions on these receptors is absent, at least on short time intervals. Apparently some other currents (or channels) affected by ammonia may underlie the mechanisms responsible for the acceleration of neuronal circuits studied in our experiments.

Certainly neural networks grown on flat surfaces in cultures *in vitro*, might have some features distinct from those for cells packaged within the tissue in closed space of scalp and the results obtained must have some limitations in their interpretation. Nevertheless such simplified systems, devoid of the complexity of whole organism, may be useful for the study of the mechanisms of toxic action of ammonia, which are yet not studied properly.

### Preserved negative feedback control and potential neuroprotective strategies

Obtained results demonstrate that negative feedback control of neural networks, based on functioning and activation of ionotropic GABA(A) and glycine receptors (Fig 8C) and of metabotropic pre- and/or postsynaptic inhibitory receptors (of type II glutamate, α2-adrenergic, CB1-cannabinoid, etc.) (Fig 9) is preserved at hyperammonemic conditions. Acceleration of neuronal networks by ammonium ions may be effectively damped up by the application of glycine (Fig 8C), by the agonists of inhibitory metabotropic receptors (Fig 9), as effectively as it is inhibited by the antagonists of NMDA (Fig 8A) and AMPA/kainate receptors (Fig 8B).

L-arginine, L-carnitine, acetyl-L-carnitine and betaine also demonstrate visible neuroprotective effects, suppressing the activities of networks activated by ammonium ions (Fig 10).

Not excluding possible action of methylated compounds on cholinergic muscarinic receptors, betaine and related compounds presumably may tonically suppress (brake) accelerated networks, by modulating the activities of GABA receptors or GABA level in the interstitial space (Figs 10 and 11). Finally all this means that various agonists (natural chemical compounds) related for example to classes of guanidines and methylamines (Figs 10 and 11), might be used to strengthen negative feedbacks and to slow down (or to brake) neuronal networks accelerated by ammonium ions.

## Conclusions

These results provide the background for the idea that primary and secondary toxic effects of ammonium ions, related to over activation of NMDA receptors (by yet unknown mechanism) and modulation of other yet unknown targets in neural networks, may be effectively prevented by reinforcing feedback control of neural networks, based on functioning of various inhibitory receptors in the system (i.e. by braking of the networks).

## Supporting Information

S1 Fig NH_4_Cl induces changes in intracellular pH in pepresentative neuron in network. Neuronal culture 12 DIV. Total number of cells involved into network is 112. **(A)** The record describing typical response of representative cell (one of 92% cells in the network) on application of 6 mM NH_4_Cl. **(B)** The bars indicate the average amplitudes ± SD of intracellular pH in 23 cells recorded at time-points indicated as (I), (II), (III). * *P* = 0.05. ** *P* = 0.01.
(TIF)

S2 Fig Typical neuronal Ca^2+^ responses evoked by the application of 35 mM KCl. Neuronal culture 16 DIV, corresponds to culture presented on Fig 6. Total number of cells involved into network is 106. n = 5. The records represent four most typical response of neuronal cells in the network to short-term (25 s) test application of 35 mM KCl. Curves from 1 through 4 describe typical responses of 12, 36, 45 and 7% of monitored cells, correspondingly. The effect varies within the limits of 5–10% from one culture to another.
(TIF)

S3 Fig Inhibitory effect of betaine disappears in the presence of acetyl-L-carnitine. Culture 15 DIV. The record of representative cell. All other abbreviations and descriptions as on Fig 10. N = 123. n = 4. 200 nM of L-glutamate was added before application of NH_4_Cl. Added acetyl-L-carnitine (10 mM) cancels inhibitory effect of betaine (10 mM) on the network accelerated by 8mM NH_4_Cl.
(TIF)

S4 Fig Inhibitory effects of L-carnitine and betaine on the stimulation of neuronal networks by ammonium ions are observed in the presence of muscarinic receptors antagonists. Cultures 15–16 DIV. The records of representative cells. All other abbreviations and descriptions as on Fig 10. **(A, B)** Telensepine (100 nM) modifies inhibitory action of L-carnitine (10 mM) on the effect evoked by 8mM NH_4_Cl. L-carnitine may suppress Ca^2+^-oscillations after some delay (Fig A; the effect is observed in 50% of cultures, n = 10) or may modify and slow down them (Fig B; the effect observed in 50% of cultures used, n = 10). **(C)** Methoctramine (500 nM; Fig C) does not modify the response to L-carnitine(10 mM) of the network accelerated by 8mM NH_4_Cl. N = 89. n = 3. 200 nM of L-glutamate was added in experiment presented on Fig C. **(D**–**F)** Inhibitory effect of betaine (10 mM) on the network stimulation by 8mM NH_4_Cl, is preserved in the presence of Telensepine (100 nM; Fig D), Methoctramine (500 nM; Fig E) and p-F-HHSiD (p-Fluoro-hexahydrosila-difenidol hydrochloride) (1.25 μM; Fig F) in most of cultures used. 200 nM of L-glutamate was added in experiment presented on Fig C. N = 98, 112, 126 for Figs from **D** to **F**, correspondingly. n = 3 for each experiment.
(TIF)

## References

[1] Sherlock S. Hepatic coma. Gastroenterology. Official publication of the American Gastroenterological Association. 1961;07: 1–8.

[2] Colombo JP. Congenital disorders of the urea cycle and ammonia detoxication. [Review]. Monogr Paediatr. 1971;1: 1–150. 4946766

[3] Hindfelt B, Siesjo BK. Cerebral effects of acute ammonia intoxication. II. The effect upon energy metabolism. Scand J Clin Lab Invest. 1971;28: 365–74. 4332525 10.3109/00365517109095711

[4] Hawkins RA, Miller AL, Nielsen RC, Veech RL. The acute action of ammonia on rat brain metabolism in vivo. Biochem J. 1973;134: 1001–8. 4762748 10.1042/bj1341001 PMC1177908

[5] O'Connor JE, Costell M, Grisolia S. Protective effect of L-carnitine on hyperammonemia. FEBS Lett. 1984;166: 331–4. 6692930 10.1016/0014-5793(84)80106-4

[6] Hahn M, Massen O, Nencki M, Pawlow J. Die Ecksche Fistel zwi der unteren Hohlvene und der Pfortader und ihre Folgen fur den Organismus. Arch Exp Pathol Pharmakol. 1893;32: 161–210. German.

[7] Nencki M, Zaleski J. Ueber die Bestimmung des Ammoniaks in Thierischen Fluessigkeiten und Geweben. Arch Exp Pathol Pharmakol. 1895;36: 385–396. German.

[8] Nencki M, Pawlow JP, Zaleski J. Ueber den Ammoniakgehalt des Bluttes und der Organe. Die Harnstoffbildung bei den Saugetieren. Arch Exp Pathol Pharmakol. 1896;37: 26–51. German

[9] Eck NV. K voprosu o perevyazkie vorotnoij veni: Predvaritelnoye soobshtshjenye. Voen Med J. 1877;130: 1–2. Russian.

[10] McDermott WV Jr. The role of ammonia intoxication in hepatic coma. Bull N Y Acad Med. 1958;34: 357–65. 13536653 PMC1805963

[11] Zuidema GD, Kirsh MM, Gaisford WD. Hepatic encephalopathy. [Review]. Major Probl Clin Surg. 1964;1: 102–26. 4950263

[12] Al Sibae MR, McGuire BM. Current trends in the treatment of hepatic encephalopathy. Ther Clin Risk Manag. 2009;5: 617–26. 19707277 10.2147/tcrm.s4443 PMC2724191

[13] Rose CF. Ammonia-lowering strategies for the treatment of hepatic encephalopathy. Clin Pharmacol Ther. 2012;92: 321–31. 10.1038/clpt.2012.112 22871998

[14] Sturgeon JP, Shawcross DL. Recent insights into the pathogenesis of hepatic encephalopathy and treatments. Expert Rev Gastroenterol Hepatol. 2014; 8: 83–100. 10.1586/17474124.2014.858598 24236755

[15] Kircheis G, Wettstein M, Dahl Sv, Haussinger D. Clinical efficacy of L-ornithine-Laspartate in the management of hepatic encephalopathy. [Review]. Metab Brain Dis. 2002;17: 453–62. 12602521 10.1023/a:1021934607762

[16] Warren KS, Schenker S. Effect of an inhibitor of glutamine synthesis (methionine sulfoximine) on ammonia toxicity and metabolism. J Lab Clin Med. 1964;64: 442–9. 14215460

[17] Brusilow SW, Traystman R. Hepatic encephalopathy. N Engl J Med. 1986;314: 786–7. 15603053

[18] Basile AS, Jones EA, Skolnick P. The pathogenesis and treatment of hepatic encephalopathy: evidence for the involvement of benzodiazepine receptor ligands. Pharmacol Rev. 1991;43: 27–71. 1674609

[19] Kosenko EA, Kaminsky YG, Korneev VN, Luk'ianova LD. Protective effect of M- and N-cholinergic receptor blockaders during acute ammonia poisoning. Bull Exp Biol Med. 1995b; 120:489–92. Russian. 8713325

[20] Nguyen JH. Blood-brain barrier in acute liver failure. Neurochem Int. 2012;60: 676–83. 10.1016/j.neuint.2011.10.012 22100566 PMC3302955

[21] Braissant O, McLin VA, Cudalbu C. Ammonia toxicity to the brain. J Inherit Metab Dis. 2013;36: 595–612. 10.1007/s10545-012-9546-2 23109059

[22] Felipo V. Hepatic encephalopathy: effects of liver failure on brain function. [Review]. Nat Rev Neurosci. 2013;14: 851–8. 10.1038/nrn3587 24149188

[23] Bosoi CR, Zwingmann C, Marin H, Parent-Robitaille C, Huynh J, Tremblay M, et al Increased brain lactate is central to the development of brain edema in rats with chronic liver disease. J Hepatol. 2014;60: 554–60. 10.1016/j.jhep.2013.10.011 24512824

[24] Brusilow SW, Koehler RC, Traystman RJ, Cooper AJ. Astrocyte glutamine synthetase: importance in hyperammonemic syndromes and potential target for therapy. Neurotherapeutics. 2010;7: 452–70. 10.1016/j.nurt.2010.05.015 20880508 PMC2975543

[25] Marcaida G, Felipo V, Hermenegildo C, Miñana MD, Grisolía S. Acute ammonia toxicity is mediated by the NMDA type of glutamate receptors. FEBS Lett. 1992;296: 67–8. 1346118 10.1016/0014-5793(92)80404-5

[26] Cauli O, Gonzalez-Usano A, Cabrera-Pastor A, Gimenez-Garzo C, Lopez-Larrubia P, Ruiz-Sauri A, et al Blocking NMDA receptors delays death in rats with acute liver failure by dual protective mechanisms in kidney and brain. Neuromolecular Med. 2014;16: 360–75. 10.1007/s12017-013-8283-5 24338618

[27] Kosenko E, Kaminsky Y, Grau E, Minana MD, Grisolia S, Felipo V. Nitroarginine, an inhibitor of nitric oxide synthetase, attenuates ammonia toxicity and ammonia-induced alterations in brain metabolism. Neurochem Res. 1995a;20: 451–6. 7544446 10.1007/BF00973101

[28] Chu CJ, Chang CC, Wang TF, Lee FY, Chang FY, Chen YC, et al Detrimental effects of nitric oxide inhibition on hepatic encephalopathy in rats with thioacetamide-induced fulminant hepatic failure: role of nitric oxide synthase isoforms. J Gastroenterol Hepatol. 2006;21: 1194–9. 16824075 10.1111/j.1440-1746.2006.04310.x

[29] Butterworth RF. Altered glial-neuronal crosstalk: cornerstone in the pathogenesis of hepatic encephalopathy. Neurochem Int. 2010;57: 383–8. 10.1016/j.neuint.2010.03.012 20350577

[30] Häussinger D, Görg B. Interaction of oxidative stress, astrocyte swelling and cerebral ammonia toxicity. Curr Opin Clin Nutr Metab Care. 2010;13: 87–92. 10.1097/MCO.0b013e328333b829 19904201

[31] Jayakumar AR, Rama Rao KV, Tong XY, Norenberg MD. Calcium in the mechanism of ammonia-induced astrocyte swelling. J Neurochem. 2009;10: 252–7. 10.1111/j.1471-4159.2009.05842.x PMC4737088 19393035

[32] Jayakumar AR, Tong XY, Curtis KM, Ruiz-Cordero R, Abreu MT, Norenberg MD. Increased toll-like receptor 4 in cerebral endothelial cells contributes to the astrocyte swelling and brain edema in acute hepatic encephalopathy. J Neurochem. 2014;128: 890–903. 10.1111/jnc.12516 24261962 PMC3951576

[33] Haack N, Dublin P, Rose CR. Dysbalance of astrocyte calcium under hyperammonemic conditions. PLoS One. 2014;9: e105832 10.1371/journal.pone.0105832 25153709 PMC4143319

[34] Ahboucha S, Gamrani H, Baker G. GABAergic neurosteroids: the "endogenous benzodiazepines" of acute liver failure. Neurochem Int. 2012;60: 707–14. 10.1016/j.neuint.2011.10.003 22041164

[35] Rangroo Thrane V, Thrane AS, Wang F, Cotrina ML, Smith NA, Chen M, et al Ammonia triggers neuronal disinhibition and seizures by impairing astrocyte potassium buffering. Nat Med. 2013;19: 1643–8. 10.1038/nm.3400 24240184 PMC3899396

[36] Lux HD. Ammonium and chloride extrusion: hyperpolarizing synaptic inhibition in spinal motoneurons. Science. 1971;173: 555–7. 5564046 10.1126/science.173.3996.555

[37] Raabe W, Gumnit RJ. Disinhibition in cat motor cortex by ammonia. J Neurophysiol. 1975;38: 347–55. 1127446 10.1152/jn.1975.38.2.347

[38] Nicoll RA. The blockade of GABA mediated responses in the frog spinal cord by ammonium ions and furosemide. J Physiol. 1978;283: 121–32. 722571 10.1113/jphysiol.1978.sp012491 PMC1282768

[39] Iles JF, Jack JJ. Ammonia: assessment of its action on postsynaptic inhibition as a cause of convulsions. Brain. 1980;103: 555–78. 7417779 10.1093/brain/103.3.555

[40] Snodgrass SR. Myoclonus: analysis of monoamine, GABA, and other systems. [Review]. FASEB J. 1990;4: 2775–88. 2165012 10.1096/fasebj.4.10.2165012

[41] Izumi Y, Svrakic N, O'Dell K, Zorumski CF. Ammonia inhibits long-term potentiation via neurosteroid synthesis in hippocampal pyramidal neurons. Neuroscience. 2013;233: 166–73. 10.1016/j.neuroscience.2012.12.035 23276672 PMC3578010

[42] Gonzalez-Usano A, Cauli O, Agusti A, Felipo V. Hyperammonemia alters the modulation by different neurosteroids of the glutamate-nitric oxide-cyclic GMP pathway through NMDA- GABAA—or sigma receptors in cerebellum in vivo. J Neurochem. 2013;125: 133–43. 10.1111/jnc.12119 23227932

[43] Schwarz CS, Ferrea S, Quasthoff K, Walter J, Gorg B, Haussinger D, et al Ammonium chloride influences in vitro-neuronal network activity. Exp Neurol. 2012;235: 368–73. 10.1016/j.expneurol.2012.02.019 22421534

[44] Kononov AV, Ivanov SV, Sokolov PA, Zinchenko VP, Dynnik VV. On the activation of neuronal networks by ammonia In: Zinchenko VP, Berezhnov AV, editors. Receptors and intracellular signaling. Pushchino: ED V. Ema; 2013 pp. 275–80. Russian.

[45] Brewer GJ, Torricelli JR, Evege EK, Price PJ. Optimized survival of hippocampal neurons in B27-Supplemental neurobasal medium, a new serum free medium combination. J Neurosci Res. 1993;35: 567–76. 8377226 10.1002/jnr.490350513

[46] Turovskaya MV, Turovsky EA, Zinchenko VP, Levin SG, Godukhin OV. Interleukin-10 modulates [Ca^2+^]_i_ response induced by repeated NMDA receptor activation with brief hypoxia through inhibition of InsP3-sensitive internal stores in hippocampal neurons. Neurosci Lett. 2012;516: 151–55. 10.1016/j.neulet.2012.03.084 22498075

[47] Darbon P, Pignier C, Niggli E, Streit J. Involvement of calcium in rhythmic activity induced by disinhibition in cultured spinal cord networks. J Neurophysiol. 2002a;88: 1461–8. 12205166 10.1152/jn.2002.88.3.1461

[48] Kelly T, Church J. Relationships between calcium and pH in the regulation of the slow afterhyperpolarization in cultured rat hippocampal neurons. J Neurophysiol. 2006;96: 2342–53. 16885515 10.1152/jn.01269.2005

[49] Onativia J, Schultz SR, Dragotti PL. A finite rate of innovation algorithm for fast and accurate spike detection from two-photon calcium imaging. J Neural Eng. 2013;10: 046017 10.1088/1741-2560/10/4/046017 23860257 PMC4038919

[50] Lin BJ, Chen TW, Schild D. Cell type-specific relationships between spiking and [Ca2+]_i_ in neurons of the Xenopus tadpole olfactory bulb. J Physiol. 2007;582: 163–75. 17463049 10.1113/jphysiol.2006.125963 PMC2075311

[51] Masuda A, Oyamada M, Nagaoka T, Tateishi N, Takamatsu T. Regulation of cytosol-nucleus pH gradients by K+/H+ exchange mechanism in the nuclear envelope of neonatal rat astrocytes. Brain Res. 1998;807: 70–7. 9756998 10.1016/s0006-8993(98)00737-9

[52] Tankanag A.V. Applications of the adaptive wavelet transform for analyzing peripheral blood flow oscillations in the human skin In: Balcerzyk M., Editor. Medical Physics. New-York: Nova Science Publishers; 2013 pp. 85–104.

[53] Granata AR. Effects of gamma-aminobutyric acid on putative sympatho-excitatory neurons in the rat rostral ventrolateral medulla in vitro. Intracellular study. Neurosci Lett. 2001;300: 49–53. 11172937 10.1016/s0304-3940(00)01673-6

[54] Pflieger JF, Clarac F, Vinay L. Picrotoxin and bicuculline have different effects on lumbar spinal networks and motoneurons in the neonatal rat. Brain Res. 2002;935: 81–86. 12062476 10.1016/s0006-8993(02)02469-1

[55] Vida I, Bartos M, Jonas P. Shunting inhibition improves robustness of gamma oscillations in hippocampal interneuron networks by homogenizing firing rates. Neuron. 2006;49: 107–17. 16387643 10.1016/j.neuron.2005.11.036

[56] Kelly T, Kafitz KW, Roderigo C, Rose CR. Ammonium-evoked alterations in intracellular sodium and pH reduce glial glutamate transport activity. Glia. 2009;57: 921–34. 10.1002/glia.20817 19053055

[57] Rose C, Kresse W, Kettenmann H. Acute insult of ammonia leads to calcium-dependent glutamate release from cultured astrocytes, an effect of pH. J Biol Chem. 2005;280: 20937–44. 15802262 10.1074/jbc.M412448200

[58] Darbon P, Scicluna L, Tscherter A, Streit J. Mechanisms controlling bursting activity induced by disinhibition in spinal cord networks. Eur J Neurosci. 2002b;15: 671–83. 11886448 10.1046/j.1460-9568.2002.01904.x

[59] Staley KJ, Soldo BL, Proctor WR. Ionic mechanisms of neuronal excitation by inhibitory GABAA receptors. Science. 1995;269: 977–81. 7638623 10.1126/science.7638623

[60] Xu TL, Gong N. Glycine and glycine receptor signaling in hippocampal neurons: diversity, function and regulation. Prog Neurobiol. 2010;91: 349–61. 10.1016/j.pneurobio.2010.04.008 20438799

[61] Langer SZ. Presynaptic autoreceptors regulating transmitter release. [Review]. Neurochem Int. 2008;52: 26–30. 17583385 10.1016/j.neuint.2007.04.031

[62] Hayashi Y, Momiyama A, Takahashi T, Ohishi H, Ogawa-Meguro R, Shigemoto R, et al Role of a metabotropic glutamate receptor in synaptic modulation in the accessory olfactory bulb. Nature. 1993;366: 687–90. 7903116 10.1038/366687a0

[63] Manzoni OJ, Castillo PE, Nicoll RA. Pharmacology of metabotropic glutamate receptors at the mossy fiber synapses of the guinea pig hippocampus. Neuropharmacology. 1995;34: 965–71. 8532177 10.1016/0028-3908(95)00060-j

[64] Imre G. The preclinical properties of a novel group II metabotropic glutamate receptor agonist LY379268. CNS Drug Rev. 2007;13: 444–64. 18078428 10.1111/j.1527-3458.2007.00024.x PMC6494167

[65] Patil ST, Zhang L, Martenyi F, Lowe SL, Jackson KA, Andreev BV, et al Activation of mGlu2/3 receptors as a new approach to treat schizophrenia: a randomized Phase 2 clinical trial. Nat Med. 2007;13: 1102–7. 17767166 10.1038/nm1632

[66] Stauffer VL, Baygani SK, Kinon BJ, Krikke-Workel JO. A short-term, multicenter, placebo-controlled, randomized withdrawal study of a metabotropic glutamate 2/3 receptor agonist using an electronic patient-reported outcome device in patients with schizophrenia. J Clin Psychopharmacol. 2014;34: 552–8. 10.1097/JCP.0000000000000187 25006819 PMC4165473

[67] Wroblewska B, Wroblewski JT, Pshenichkin S, Surin A, Sullivan SE, Neale JH. N-acetylaspartylglutamate selectively activates mGluR3 receptors in transfected cells. J Neurochem. 1997;69: 174–81. 9202308 10.1046/j.1471-4159.1997.69010174.x

[68] Bergeron R, Coyle JT, Tsai G, Greene RW. NAAG reduces NMDA receptor current in CAI hippocampal pyramidal neurons of acute slices and dissociated neurons. Neuropsychopharmacology. 2005;30: 7–16. 15354184 10.1038/sj.npp.1300559

[69] Neale SA, Salt TE. Modulation of GABAergic inhibition in the rat superior colliculus by a presynaptic group II metabotropic glutamate receptor. J Phisiol. 2006;577: 659–669. 10.1113/jphysiol.2006.119073 PMC1890443 16973709

[70] Walder KK, Ryan SB, Bzdega T, Olszewski RT, Neale JH, Lindgren CA. Immunohistological and electrophysiological evidence that N-acetylaspartylglutamate is a co-transmitter at the vertebrate neuromuscular junction. Eur J Neurosci. 2013;37: 118–29. 10.1111/ejn.12027 23134476 PMC3538924

[71] Fricker AC, Mok MH, de la Flor R, Shah AJ, Woolley M, Dawson LA, Kew JN. Effects of N-acetylaspartylglutamate (NAAG) at group II mGluRs and NMDAR. Neuropharmacology. 2009;56: 1060–7. 10.1016/j.neuropharm.2009.03.002 19285517

[72] Sanchez-Blazquez P, Rodriguez-Munoz M, Garzon J. The cannabinoid receptor 1 associates with NMDA receptors to produce glutamatergic hypofunction: implications in psychosis and schizophrenia. Front Pharmacol. 2014;4: 169 10.3389/fphar.2013.00169 24427139 PMC3877778

[73] Mitchell VA, Jeong HJ, Drew GM, Vaughan CW. Cholecystokinin exerts an effect via the endocannabinoid system to inhibit GABAergic transmission in midbrain periaqueductal gray. Neuropsychopharmacology. 2011;36: 1801–10. 10.1038/npp.2011.59 21525858 PMC3154098

[74] Wang M, Ramos BP, Paspalas CD, Shu Y, Simen A, Duque A, et al Alpha2A-adrenoceptors strengthen working memory networks by inhibiting cAMP-HCN channel signaling in prefrontal cortex. Cell. 2007;129: 397–410. 17448997 10.1016/j.cell.2007.03.015

[75] Arnsten AF, Jin LE. Guanfacine for the treatment of cognitive disorders: a century of discoveries at Yale. Yale J Biol Med. 2012;85: 45–58. 22461743 PMC3313539

[76] Calligaris L, Vidoni A, Bruno I, Vidoni M, Barbi E. Efficacy of clonidine in hyperammonemia induced hyperexcitability syndrome. Paediatr Anaesth. 2013;23: 202–4. 10.1111/pan.12088 23289776

[77] Gerova M, Torok J. Hypotensive effect of arginine metabolite, is affected by NO synthase. Phisiol Res. 2004;53: 357–363. 15311993

[78] Turovsky EA, Turovskaya MV, Berezhnov AV, Tolmacheva AV, Kaimachnikov NP, Dolgacheva LP, et al Convergence of Ca^2+^ Signaling Pathways in Adipocytes. The Role of L-Arginine and Protein Kinase G in Generation of Transient and Periodic Ca^2+^ Signals. Biochem (Mosc) Suppl Ser A Membr Cell Biol. 2012;6: 35–44.

[79] Kloiber O, Banjac B, Drewes LR. Protection against acute hyperammonemia: the role of quaternary amines. Toxicology. 1988;49: 83–90. 2897729 10.1016/0300-483x(88)90178-3

[80] O'Connor JE, Costell M. New roles of carnitine metabolism in ammonia cytotoxicity. Adv Exp Med Biol. 1990;272: 183–95. 2103686 10.1007/978-1-4684-5826-8_12

[81] Falchetto S, Kato G, Provini L. The action of carnitines on cortical neurons. Can J Physiol Pharmacol. 1971;49: 1–7. 5572414 10.1139/y71-001

[82] Janiri L, Falcone M, Persico A, Tempesta E. Activity of L-carnitine and L-acetylcarnitine on cholinoceptive neocortical neurons of the rat in vivo. J Neural Transm Gen Sect. 1991;86: 135–46. 1683239 10.1007/BF01250574

[83] Minana MD, Hermenegildo C, Llsansola M, Montoliu C, Grisolia S, Felipo V. Carnitine and choline derivatives containing a trimethylamine group prevent ammonia toxicity in mice and glutamate toxicity in primary cultures of neurons. J Pharmacol Exp Ther. 1996;279: 194–201. 8858993

[84] Warskulat U, Reinen A, Grether-Beck S, Krutmann J, Haussinger D. The osmolyte strategy of normal human keratinocytes in maintaining cell homeostasis. J Invest Dermatol. 2004;123: 516–21. 15304091 10.1111/j.0022-202X.2004.23313.x

[85] Fisher SK, Heacock AM, Keep RF, Foster DJ. Receptor regulation of osmolyte homeostasis in neural cells. J Physiol. 2010;588: 3355–64. 10.1113/jphysiol.2010.190777 20498228 PMC2988502

[86] Turovsky EA, Turovskaya MV, Dolgacheva LP, Zinchenko VP, Dynnik VV. Acetylcholine Promotes Ca^2+^ and NO-Oscillations in Adipocytes Implicating Ca^2+^→NO→cGMP→cADP-ribose→Ca^2+^ Positive Feedback Loop—Modulatory Effects of Norepinephrine and Atrial Natriuretic Peptide. PLoS ONE. 2013;16: e63483. 10.1371/journal.pone.0063483 PMC3656004 23696827

[87] Smith MD, Saunders GW, Clausen RP, Frolund B, Krogsgaard-Larsen P, Larsson OM, et al Inhibition of the betaine-GABA transporter (mGAT2/BGT-1) modulates spontaneous electrographic bursting in the medial entorhinal cortex (mEC). Epilepsy Res. 2008;79: 6–13. 10.1016/j.eplepsyres.2007.12.009 18262393 PMC4314296

[88] Marchetti C, Pagnotta S, Donato R, Nistri A. Inhibition of spinal or hypoglossal motoneurons of the newborn rat by glycine or GABA. Eur J Neurosci. 2002;15: 975–83. 11918657 10.1046/j.1460-9568.2002.01927.x

[89] Butterworth RF. Pathophysiology of hepatic encephalopathy: a new look at ammonia. [Review]. Metab Brain Dis. 2002;17: 221–7. 12602499 10.1023/a:1021989230535

[90] Cauli O, Rodrigo R, Llansola M, Montoliu C, Monfort P, Piedrafita B, et al Glutamatergic and gabaergic neurotransmission and neuronal circuits in hepatic encephalopathy.Metab Brain Dis. 2009;24: 69–80. Review. 10.1007/s11011-008-9115-4 19085094

[91] Chergui K, Charlety PJ, Akaoka H, Saunier CF, Brunet JL, Buda M, et al Tonic activation of NMDA receptors causes spontaneous burst discharge of rat midbrain dopamine neurons in vivo. Eur J Neurosci. 1993;5: 137–44. 8261095 10.1111/j.1460-9568.1993.tb00479.x

[92] Legrand JC, Darbon P, Streit J. Contributions of NMDA receptors to network recruitment and rhythm generation in spinal cord cultures. Eur J Neurosci. 2004;19: 521–32. 14984403 10.1111/j.0953-816x.2003.03143.x

[93] Yue C, Yaari Y. KCNQ/M channels control spike afterdepolarization and burst generation in hippocampal neurons. J Neurosci. 2004;24: 4614–24. 15140933 10.1523/JNEUROSCI.0765-04.2004 PMC6729392

[94] Goldberg JA, Wilson CJ. Control of spontaneous firing patterns by the selective coupling of calcium currents to calcium-activated potassium currents in striatal cholinergic interneurons. J Neurosci. 2005;25: 10230–8. 16267230 10.1523/JNEUROSCI.2734-05.2005 PMC1343481

[95] Lee CR, Tepper JM. A calcium-activated nonselective cation conductance underlies the plateau potential in rat substantia nigra GABAergic neurons. J Neurosci. 2007;27: 6531–41. 17567814 10.1523/JNEUROSCI.1678-07.2007 PMC6672447

[96] Kleiman-Weiner M, Beenhakker MP, Segal WA, Huguenard JR. Synergistic roles of GABAA receptors and SK channels in regulating thalamocortical oscillations. J Neurophysiol. 2009;102: 203–13. 10.1152/jn.91158.2008 19386752 PMC2712277

[97] Kelly T, Church J. The weak bases NH(3) and trimethylamine inhibit the medium and slow afterhyperpolarizations in rat CA1 pyramidal neurons. Pflugers Arch. 2005;451: 418–27. 16047153 10.1007/s00424-005-1483-6

[98] Hertz L, Peng L, Song D. Ammonia, like K(+), stimulates the Na(+), K(+), 2 Cl(-) cotransporter NKCC1 and the Na(+),K(+)-ATPase and interacts with endogenous ouabain in astrocytes. Neurochem Res. 2015;40: 241–57. 10.1007/s11064-014-1352-9 24929663

[99] Wang F, Du T, Liang C, Verkhratsky A, Peng L. Ammonium increases Ca^2+^ signalling and upregulates expression of Ca_v_ 1.2 gene in astrocytes in primary cultures and in the in vivo brain. Acta Physiol (Oxf). 2015; In press. 10.1111/apha.12500 25846713

